# MiR-3529-3p from PDGF-BB-induced cancer-associated fibroblast-derived exosomes promotes the malignancy of oral squamous cell carcinoma

**DOI:** 10.1007/s12672-023-00753-9

**Published:** 2023-09-05

**Authors:** Dingyun You, Yanghao Wang, Jianguo Xu, Rongqiang Yang, Weizhou Wang, Xiaofang Wang, Xue Cao, Yiting Li, Lifu Yu, Weihong Wang, Yanan Shi, Changbin Zhang, Hefeng Yang, Yongwen He, Li Bian

**Affiliations:** 1 Department of Dental Research, The Affiliated Stomatological Hospital of Kunming Medical University, Kunming, 650106 Yunnan China; 2 The Yunnan Key Laboratory of Stomatological, Kunming Medical University, Kunming, 650106 Yunnan China; 3 Department of Pathology, The First Affiliated Hospital of Kunming Medical University, Kunming, 650032 Yunnan China; 4 Department of Orthopaedics, The First Affiliated Hospital of Kunming Medical University, Kunming, 650032 Yunnan China; 5 Department of Pathology, The Second Affiliated Hospital of Kunming Medical University, Kunming, 650032 Yunnan China; 6 Department of Laboratory Animal Science, Kunming Medical University, Kunming, 650500 Yunnan China; 7 Department of Oral and Maxillofacial Surgery, The Affiliated Stomatological Hospital of Kunming Medical University, Kunming, 650106 Yunnan China; 8 Department of Dental Research, Qujing Medical College, Qujing, 655011 Yunnan China

**Keywords:** OSCC, Exosomes, miR-3529-3p, PDGF-BB, Cancer-associated fibroblasts

## Abstract

**Aims:**

This study aims to explore the role of exosomes from cancer-associated fibroblasts (CAFs) induced by PDGF-BB in promoting the malignancy of oral squamous cell carcinoma (OSCC) and provide new insight into the mechanism of OSCC progression and its treatment.

**Main methods:**

Exosomes were extracted from human oral mucosa fibroblasts (hOMFs) and CAFs. Differentially expressed miRNAs of exosomes between hOMFs and CAFs were analysed using high-throughput sequencing and self-programmed R software. Cal-27, a human tongue squamous carcinoma cell line, was treated with exosomes. Differentially expressed miRNAs between clinical cancer tissues and adjacent tissues and between hOMF and CAF exosomes were verified by qRT‒PCR. The effect of miR-3529-3p on Cal-27 cells was clarified by overexpressing or knocking down miR-3529-3p in Cal-27 cells. Sample expression and differentially expressed miRNA expression were compared between cancer and paracarcinoma tissues.

**Key findings:**

We found that exosomes from CAFs (CAF-Exos) were internalized by tongue squamous carcinoma cells and promoted their proliferation, migration, invasion, and antiapoptotic effects. MiR-3529-3p was a significant differentially expressed miRNA between CAF-Exos and exosomes from hOMFs (hOMF-Exos). The overexpression of miR-3529-3p promoted proliferation, migration, and invasion and inhibited apoptosis of Cal-27 cells.

**Significance:**

This study explores the role of PDGF-BB-induced CAFs in promoting malignancy in OSCC. This study will provide new insight into the mechanism of OSCC progression and its treatment.

**Supplementary Information:**

The online version contains supplementary material available at 10.1007/s12672-023-00753-9.

## Introduction

Oral squamous cell carcinoma (OSCC) is the sixth most common malignancy worldwide. This disease severely affects the appearance, chewing, swallowing, talking, and other functions of patients [1]. There are more than 600,000 new cases of OSCC and 300,000 deaths worldwide each year, and the trend is increasing every year [2]. The current 5-year survival rate for OSCC has been maintained at 50% [3, 4]. The tumour microenvironment plays important roles in immunosuppression, distant metastasis, local drug resistance, and targeted therapy. Carcinoma-associated fibroblasts (CAFs) have received increasing attention in recent years as an important component of the tumour microenvironment [5]. Tumour progression is highly dependent on CAFs and has become a new target for antitumour therapy. However, the interaction between CAFs and cancer cells remains unclear and deserves in-depth study [6, 7].

Human platelet-derived growth factor (PDGF) plays a key role in embryonic development, cell growth and differentiation and the tissue damage response. A variety of pathological processes are associated with the abnormal activity of PDGF and its receptors. In our previous study, we demonstrated that OSCC can induce the reprogramming of human oral mucosa fibroblasts (hOMFs) to cancer-associated fibroblasts (CAFs) through high secretion of platelet-derived growth factor-BB (PDGF-BB). Activated CAFs produce extracellular matrix and exosomes to support tumour growth. hOMFs can be induced to CAFs by cancer cells in vivo and have a significant tumour-promoting effect [8]. Upon PDGF-BB stimulation, hOMF cells activate the NF-κB pathway and highly express CAF markers such as α-SMA, FAP-α, PDGFR-β, and p-PDGFR-β. Activated fibroblasts can then further promote cancer cells to secrete high levels of PDGF-BB, thus forming a positive feedback loop of interactions [9]. The above studies show that PDGF-BB can induce hOMF reprogramming to CAFs, which has a coupling interaction with tumour cells and is sufficient to promote tumour progression. However, the mechanisms involved need to be further investigated.

Recent studies have revealed that carcinoma-associated fibroblast-derived exosomes (CAF-Exos) are involved in mediating the exchange of material and information between tumour cells in the tumour microenvironment and are important for cell‒cell interactions. MicroRNAs (miRNAs) play an important role in CAF-Exos [10]. A characteristic feature of CAFs compared to normal fibroblasts is the dysregulation of miRNAs. This process leads to remodelling of the tumour microenvironment and the corresponding induction of drug resistance, cell migration and invasion, and tumour cell proliferation [11, 12]. Using miRNAs or miRNA inhibitors to regulate exosomal miRNAs in CAFs is a new idea and approach in tumour therapy. Although a few studies have investigated the correlation between CAF-Exos and tumour progression, the roles of exosomes released from PDGF-BB-induced CAFs from hOMF reprogramming in OSCC malignancy have not been reported. Therefore, this topic needs more in-depth study.

## Materials and methods

### Induction and culture of CAFs

Normal human hOMFs were purchased from Qingqi Biologicals. hOMFs were routinely cultured in high-glucose DMEM containing 10% FBS at 37 °C and 5% CO_2_. CAFs were induced by treating hOMF cells with 30 ng/mL PDGF-BB for 72 h.

### Extraction and identification of exosomes

The cells at 80% confluence were rinsed with PBS three times and cultured with high glucose DMEM with 10% exosome-free foetal bovine serum for 48 h. The supernatant was collected, filtered through a 0.22 μm filter, and concentrated using 3 kDa ultrafiltration. The exosomes were extracted using the exoEasy Maxi kit. Fresh exosomes were stained using phosphotungstic acid. Their morphology was observed using a transmission electron microscope (JEOL JEM-1011). The number and diameter of exosomes were analysed using a Flow NanoAnalyzer (Xiamen Flow Biological Company).

### Quantitative real-time polymerase chain reaction (qRT‒PCR)

RNA was extracted according to the manufacturer's instructions (TRIzol Universal, Tiangen, DP424). cDNA was synthesized using the Tiangen Reverse Transcription Kit (KR116). The qRT‒PCR mixture was denatured at 95 °C for 10 min, followed by 95 °C for 10 s and 60 °C for 32 s, and this process was repeated for 40 cycles. The sequences of the primers are shown in Supplementary Table 1.

### Western blot (WB)

Cells or exosomes were lysed in RIPA buffer containing 1% PMSF, and the protein concentration was determined using the BCA method (Beyotime). Protein was separated by 10% SDS‒PAGE, transferred to PVDF membranes, and incubated with primary antibodies and HRP-coupled secondary antibodies. The membranes were subjected to ECL luminescent solution. The greyscale values were analysed using ImageJ software. Antibody information is detailed in Supplementary Table 2.

### Cellular uptake of exosomes

Exosomes were labelled using PKH26 according to the instructions. The cells (Cal-27, HSC-4, SCC-6) were cultured in confocal culture dishes, incubated with labelled hOMF-Exos, CAF-Exos, or PBS (NC group) for 6 h and 24 h, and fixed with 4% paraformaldehyde. The cells were stained with DAPI and were subjected to a laser scanning confocal microscope.

### Cell proliferation assay

The cells (Cal-27, HSC-4, SCC-6) were incubated in 96-well plates for 0 h, 24 h, 48 h, and 72 h, and a Cell Counting Kit-8 (CCK8) assay was performed.

### Wound healing assay

Cells (Cal-27, HSC-4, SCC-6) were inoculated into Culture-Insert with scratches up to 500 μm wide. The length of scratches was observed and recorded under the microscope after culturing for 0, 6, 12, and 24 h. The scratched area was quantified using ImageJ software.

### Invasion assay

The matrix gel was diluted at a ratio of 1:8, and 100 μL of the diluted matrix gel was added to the upper chamber of the Transwell plate and incubated for 30 min at 37 °C. A 200 μL cell suspension (4 × 10^4^ cells) was added to the upper chamber, and 500 μL of complete medium containing 20% FBS was added to the lower chamber. The plates were incubated for 48 h. The cells were gently wiped off the surface of the matrix gel, and the transwell was fixed with 4% paraformaldehyde and stained with 0.1% crystal violet. Six random fields were counted under a light microscope.

### Apoptosis analysis

Cells (Cal-27, HSC-4, SCC-6) at 70% confluence were treated with 2 mM H2O2 for 60 min to induce apoptosis. After induction, the cells were digested by trypsin, stained with 5 μL of Annexin V/FITC and 10 μL of PI according to the kit (Beyotime), and analysed by flow cytometry.

### Coculture model for transwell plates

The upper chamber was inoculated with hOMF cells, and the lower chamber was inoculated with Cal-27 cells. Different groups of cells were treated with the PDGFR inhibitor CP-673451 (10 nmol/mL, Selleck, S153606) and the exosome inhibitor GW4869 (1 μmol/mL, Selleck, S7609) for 48 h. CP-673451 is a selective PDGFRα/β inhibitor. GW4869 is a noncompetitive inhibitor of neutral membrane sphingomyelinase and is also a common exosome inhibitor.

### High-throughput sequencing analysis of miRNAs (miR)

RNA of 18–30 nt was purified from total RNA for library construction, and high-throughput sequencing was performed after quality testing. The data were filtered using SOAPnuke (v1.5.2) software. The sequences of the samples were aligned to the reference genome using the alignment system HISAT2 (v2.0.4). Gene expression was further calculated for differentially expressed genes between samples, which were selected based on both the fold of difference (| log2(fold change) |> 1) and the level of significance (p value < 0.05). The overall distribution of differential miRs was analysed by a heatmap.

### Clinical tumour sample collection

In this study, 26 OSCC samples were collected from the First Affiliated Hospital of Kunming Medical University in June 2020. All patients signed an informed consent form, and this study was approved by the ethics committee of the First Affiliated Hospital of Kunming Medical University. The expression of miR-3529-3p was analysed in cancer tissues and adjacent tissues and exosomes of cancer and adjacent tissues.

### Transfection of small interfering RNA (siRNA)

Mimic/inhibitor/mimic NC/inhibitor NC was purchased from Bioengineering Shanghai, Ltd. The target sequences are shown in Supplementary Table 1. Cells at 40–60% confluence were transfected with mimic/inhibitor/mimic NC/inhibitor for 48 h. Transfection efficiency was observed using fluorescence microscopy and qRT‒PCR.

### Target gene prediction of miR-3529-3p and its functional analysis

We predicted the target genes of miR-3529-3p by TargetScanHuman database (https://www.targetscan.org/vert_80/) and miRDB database (http://mirdb.org/). We used R (4.2.1) software and ggplot2 [3.3.6] to make Venn diagrams of the predicted target genes from both sites. "GO and KEGG enrichment analysis" was analyzed and visualized using the R package (version 3.6.3). The ggplot2 package and the cluster Profiler package [version 3.14.3] were used for the above analysis. The STRING website (https://string-db.org/), Cytoscape (version 3.9.0), and the MNC algorithm were applied to visualize PPI networks and screen hub genes. RNAseq data from the TCGA database (https://portal.gdc.cancer.gov) were downloaded and collated from the TCGA-OSCC (oral squamous cancer) project STAR process and extracted in TPM format as well as clinical data. Statistical analysis and visualization were performed using R software v3.6.3. survminer package [version 0.4.9] was used for visualization. survival package [version 3.2-10] was used for the statistical analysis of survival data. Statistical analysis were performed using Cox regression. *P* < 0.05 was considered statistically significant.

### Statistical analysis

All experimental data were statistically analyzed using GraphPad Prism or R software. Independent samples t tests, paired t tests, or nonparametric analysis was used when there were only two groups. One-way ANOVA was used when there were three or more groups, and the LSD-t test or Dunnett-t was used when statistically significant.

## Results

### Induction of hOMFs to CAFs using PDGF-BB and exosome extraction

**Fig. 1:**
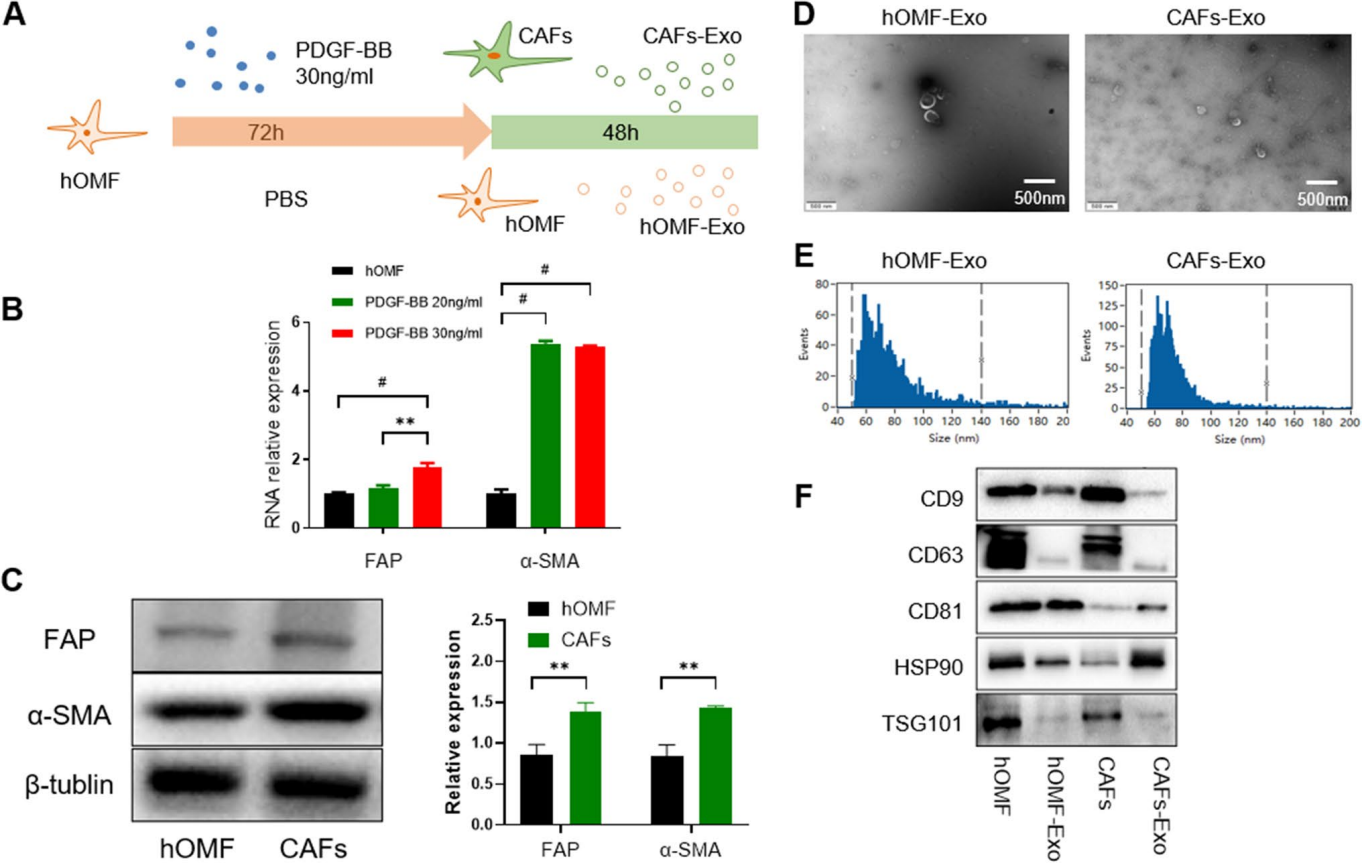
**I**nduction of hOMF to CAFs using PDGF-BB and exosome extraction. **A** Schematic diagram of CAFs induction. **B** Gene expression of α-SMA and FAP in hOMF and CAFs by qRT-PCR. **C** Protein expression levels of α-SMA and FAP in hOMF and CAFs by WB detection; **D** observation of hOMF-Exo/CAFs-Exo morphology using transmission electron microscopy; **E** flow NanoAnalyzer detection of hOMF-Exo/CAFs-Exo particle size distribution; **F** analysis of hOMF-Exo/CAFs-Exo surface markers using WB

In this study, we found that after stimulating hOMFs for 72 h using 30 ng/mL PDGF-BB (Fig. 1A), the cells remained short and spindle-shaped with no significant difference in morphology compared with those before stimulation. Western blotting (WB) and qRT‒PCR results revealed that the expression of α-SMA and FAP was significantly increased in the PDGF-BB-stimulated group compared to the hOMF group (Fig. 1B, C), suggesting that PDGF-BB successfully induced hMOFs to CAFs.

CAF-Exos/hOMF-Exos both showed a typical circular-like bilayer-like structure by transmission electron microscopy (Fig. 1D). hOMF-Exos had a mean particle size of 75.52 nm, and CAF-Exos had a mean particle size of 72.24 nm (Fig. 1E). The exosome markers CD9, CD63, CD81, HSP90 and TSG101 were positively expressed (Fig. 1F).

### CAF-Exos are internalized by OSCC cells and promote their proliferation, migration, invasion, and antiapoptotic capacity

**Fig. 2:**
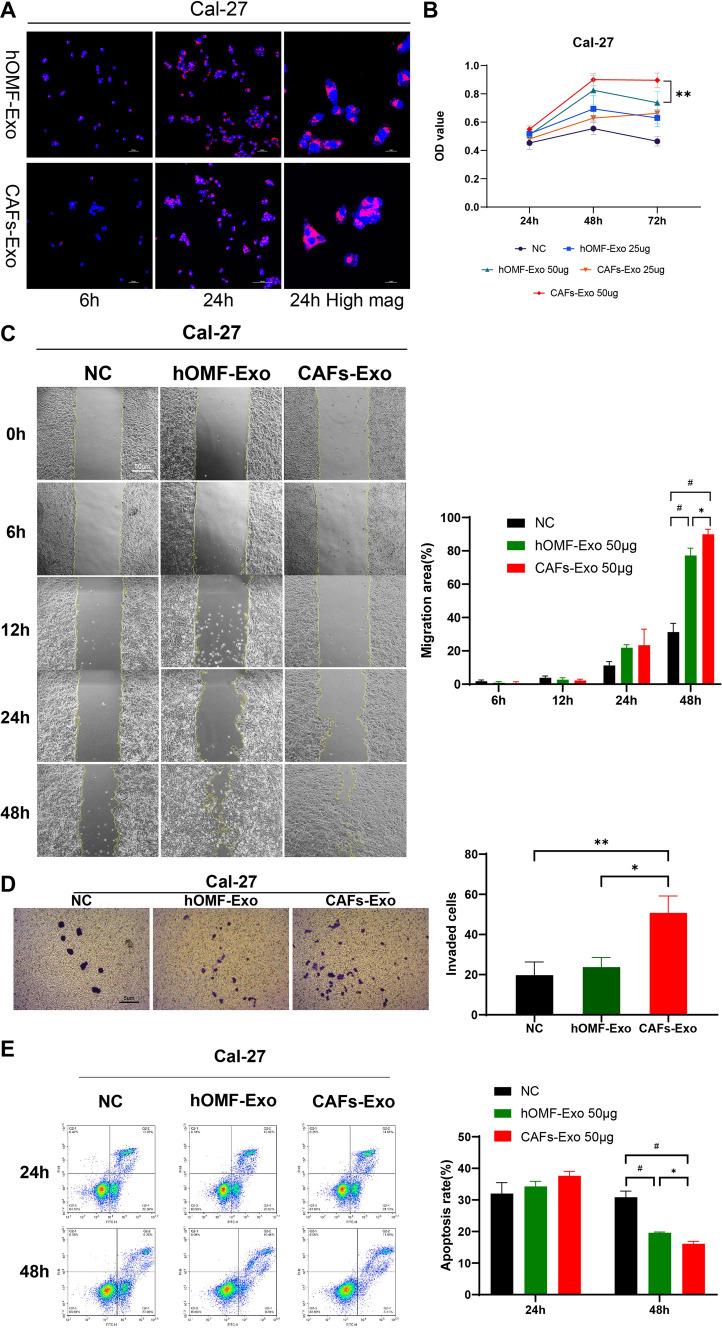
CAFs-Exo promotes proliferation, migration, invasion, and anti-apoptotic ability in tongue squamous carcinoma cells. **A** Uptake of exosomes by tongue squamous carcinoma cells, blue fluorescence is DAPI-stained nuclei and red fluorescence is PKH26-labelled hOMF-Exo/CAFs-Exo. **B** Changes in proliferation ability after 24 h, 48 h and 72 h of hOMF-Exo/CAFs-Exo treatment in Cal-27. **C** Migration of Cal-27 after 0–48 h of hOMF-Exo/CAFs-Exo treatment and quantitative analysis of migration rate. **D** Detection of invasive ability of Cal-27 after exosome treatments. **E** Apoptotic analysis of Cal-27 after hOMF-Exo/CAFs-Exo treatment for 24 h, 48 h. *P < 0.05; **P < 0.01; ^#^*P* < 0.001

Coculture of PKH26-labelled hOMF-Exos/CAF-Exos with tongue squamous carcinoma cells (Cal-27, HSC-4, SCC-6) revealed that exosomes started to accumulate around the nucleus after 6 h and increased significantly after 24 h in each cell line (Fig. 2A). These results indicated that the uptake of exosomes started at 6 h and increased with time. The proliferative capacity of each cell line was significantly enhanced after exosome intervention. High concentrations of exosomes promoted cell proliferation more strongly than low concentrations of exosomes. After 72 h of stimulation at 50 μg/mL, the proliferation of Cal-27 and HSC-4 cells increased significantly in the CAF-Exo group compared with the hOMF-Exo group (Figs. 2B, S1A). Therefore, an exosome concentration of 50 μg/mL was chosen for the subsequent experiment.

The effect of hOMF-Exos and CAF-Exos on the migration of Cal-27/HSC-4/SCC-6 cells was analysed in a wound-healing assay. The results showed that the migration of hOMF-Exo/CAF-Exo-treated OSCCs was significantly higher than that of the NC group at both 24 h and 48 h and was most obvious at 48 h (Figs. 2C, S1B). In Cal-27 cells, the cell migration rate was significantly higher in the CAF-Exo group than in the hOMF-Exo group (Fig. 2C). We also examined the invasive ability of Cal-27 cells after 48 h of exosome stimulation. The results showed that there was no significant difference in the invasive ability of cells in the hOMF-Exo group compared to the NC group. The invasive ability was significantly increased in the CAF-Exo group compared to the other two groups (Fig. 2D).

The results of apoptosis analysis showed statistically significant differences among the three groups at 24 h. However, the apoptosis of the CAF-Exo and hOMF-Exo groups was not statistically significant. Both exosomes significantly inhibited apoptosis in Cal-27 cells after 48 h, and apoptosis in the CAF-Exo group was lower than that in the hOMF-Exo group (P = 0.0305, Fig. 2E). CAF-Exos and hOMF-Exos significantly inhibited H2O2-induced apoptosis in the other two cancer cell lines (HSC-4 and SCC-6) after 48 h of stimulation. However, the antiapoptotic differences between the two exosomes were not statistically significant (Fig S1D, E).

### The proliferation, migration, invasion, and antiapoptotic capacity of Cal-27 cells can be inhibited by inhibiting the PDGF receptor or exosomes of CAFs

**Fig. 3:**
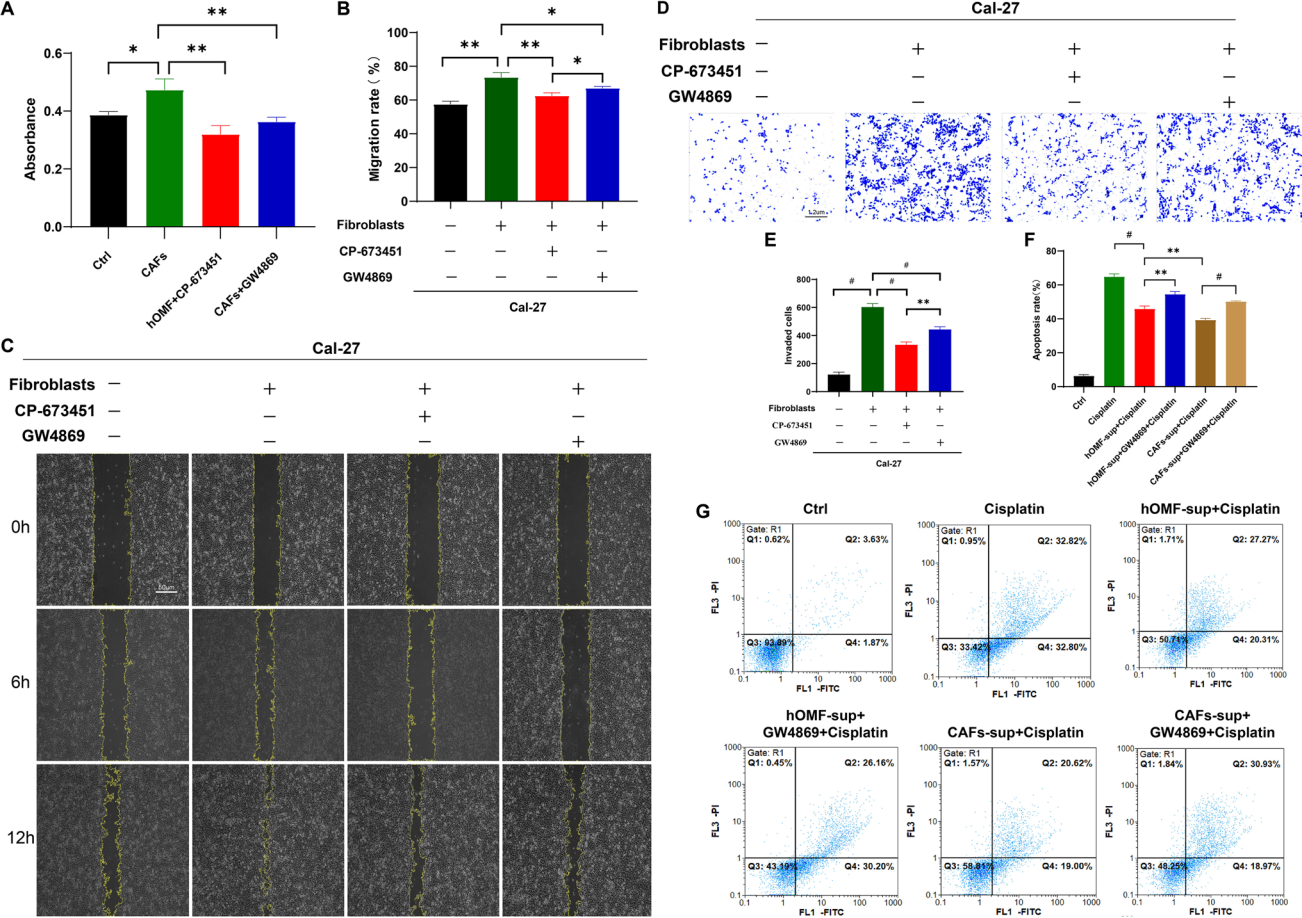
Effect of inhibition of the PDGF receptor or exosome of CAFs on the proliferation, migration, invasion and anti-apoptotic capacity of Cal-27 cells. **A** Inhibition of the PDGF receptor or exosome of CAFs effectively inhibits Cal-27 proliferation. **B**, **C** Inhibition of the PDGF receptor or exosome of CAFs effectively inhibits the migration of Cal-27 cells. **D**, **E** Inhibition of the PDGF receptors or exosomes of CAFs can effectively inhibit Cal-27 cell invasion. **F**, **G** Inhibition of PDGF receptors or exosomes of CAFs can effectively inhibit the anti-apoptotic capacity of Cal-27 cells. *P < 0.05; **P < 0.01; ^#^*P* < 0.001

We explored the cell phenotypic changes after treatment of different groups of cells with the PDGFR inhibitor CP-673451 and exosome inhibitor GW4869 using a coculture model of Transwell plates. The results of the CCK8 assay showed that cell proliferation was enhanced in the CAF+ Cal-27 group compared to the Cal-27 alone group, with statistical significance (*P* = 0.019). Cell proliferation was reduced in the hOMF + Cal-27 + CP-673451 group compared to the CAF+ Cal-27 group (*P* = 0.0053), indicating that platelet-derived growth factor receptor inhibitors inhibited the activation of hOMFs into CAFs, which in turn inhibited the proliferation of Cal-27 cells. Cal-27 cell proliferation was significantly reduced in the hOMF + Cal-27+ GW4869 group compared to the CAF+ Cal-27 group (*P* = 0.0095) (Fig. 3A).

The results of the wound healing assay showed that migration was significantly enhanced in the CAF+ Cal-27 group compared to the Cal-27 group. Compared with the hOMF + Cal-27 group, the hOMF + Cal-27 + CP-673451 group showed reduced migration, indicating that the inhibitor of platelet-derived growth factor receptor could inhibit the activation of hOMFs into CAFs, which in turn inhibited the migration of Cal-27 cells. The migration was significantly reduced in the hOMF + Cal-27 + GW4869 group compared to the hOMF + Cal-27 group (*P* = 0.0095) (Fig. 3B, C). The invasive assay results showed that the invasive ability in the CAF + Cal-27 group was significantly enhanced compared to that in the NC group. Compared with that of the hOMF + Cal-27 group, the invasion of the hOMF + Cal-27 + CP-673451 group was reduced, indicating that the inhibitor of platelet-derived growth factor receptor inhibits the activation of hOMFs into CAFs, which in turn inhibits the invasion of Cal-27 cells. The invasive ability was significantly reduced in the hOMF + Cal-27 + GW4869 group compared to the hOMF + Cal-27 group (*P* = 0.0009) (Fig. 3D, E).

In this study, the supernatants were collected after hOMFs and CAFs were treated with the exosome inhibitor GW4869 (1 μmol/mL) for 48 h. Cal-27 cells were incubated with the supernatants and cisplatin (2 mg/L) for 48 h, and apoptosis was analysed.

The results showed that the antiapoptotic ability was enhanced in the hOMF-sup + cisplatin group compared to the cisplatin group (*P* < 0.001). The antiapoptotic ability in the hOMF-sup + GW4869 + cisplatin group was reduced compared to that in the hOMF-sup + cisplatin group (*P* = 0.0033). The antiapoptotic ability of cells in the CAF-sup + cisplatin group was enhanced compared to that in the hOMF-sup + cisplatin group (*P* = 0.0051). The antiapoptotic ability was decreased in the CAF-sup + GW4869 + cisplatin group compared with the CAF-sup + cisplatin group (*P* < 0.001) (Fig. 3F, G). The above results indicated that CAF-Exos could significantly enhance the antiapoptotic ability of Cal-27 cells.

### MiR-3529-3p is differentially expressed between CAF-Exos and hOMF-Exos

**Fig. 4:**
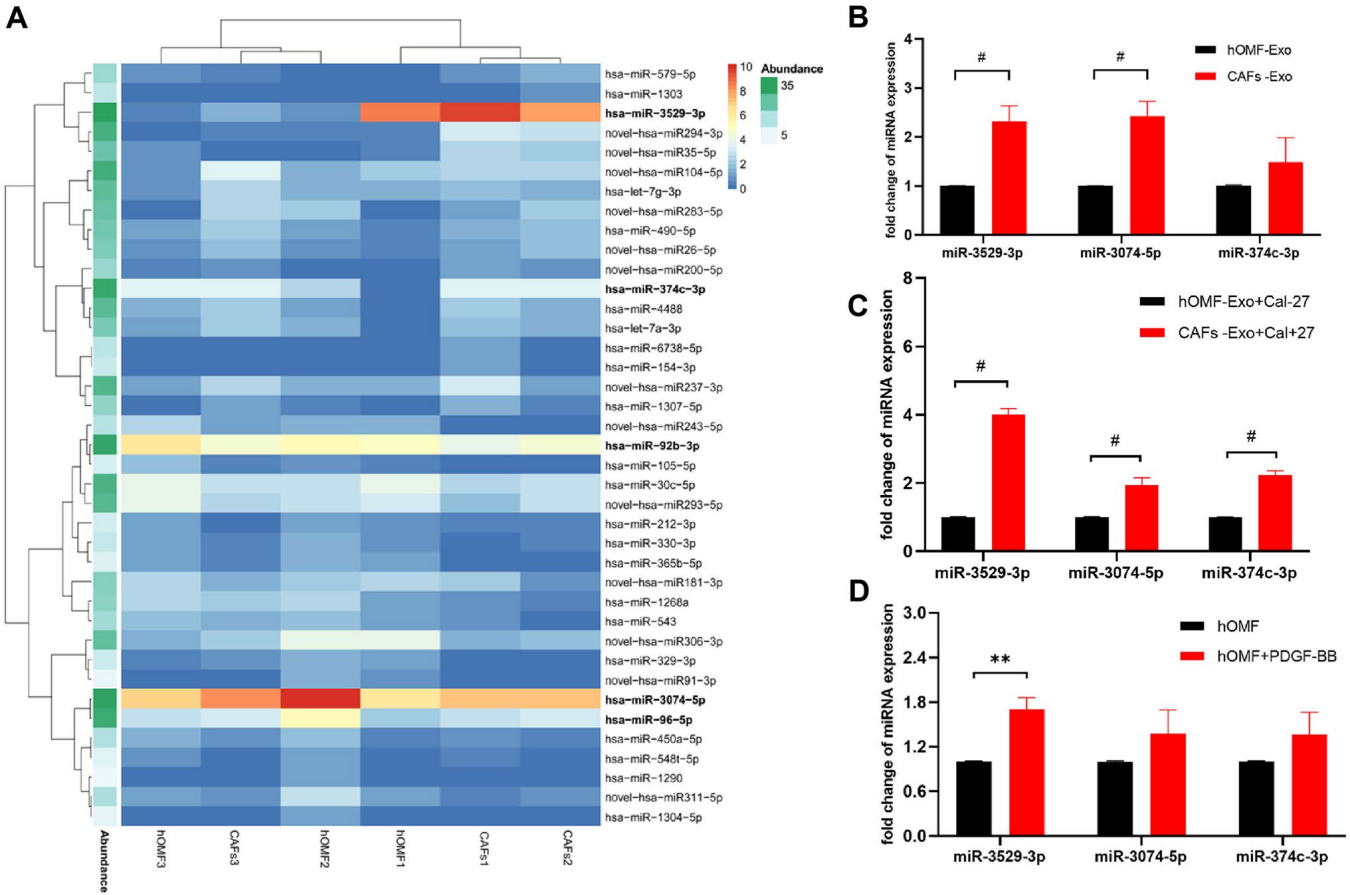
Results of differential miRNA screening and validation. **A** Differential expression of miRNAs in Heat map (horizontal coordinates are sample names, vertical coordinates are IDs of miRNAs, red indicates high expression ploidy, blue indicates low expression ploidy) **B** Expression of selected miRNAs in CAFs-Exo and hOMF-Exo; **C** Expression of selected miRNAs in Cal-27 after exosome stimulation; D: Expression of selected miRNAs in CAFs and hOMF in the validation of qRT-PCR. *P < 0.05; **P < 0.01; ^#^*P* < 0.001

A total of 39 significantly differentially expressed miRNAs were identified between CAF-Exos and hOMF-Exos in this study (Fig. 4A) (Supplementary Table 3). Given the abundance, ploidy change value, q-value of miRs and the operability of subsequent experiments conducted, we selected miRs with q-values ranking in the top 5 and higher abundance in the CAF-Exo group than in the hOMF-Exo group for validation (Supplementary Table 4). A total of three miRs were finally selected, namely, miR-3529-3p, miR-3074-5p, and miR-374c-3p.

**Fig. 5:**
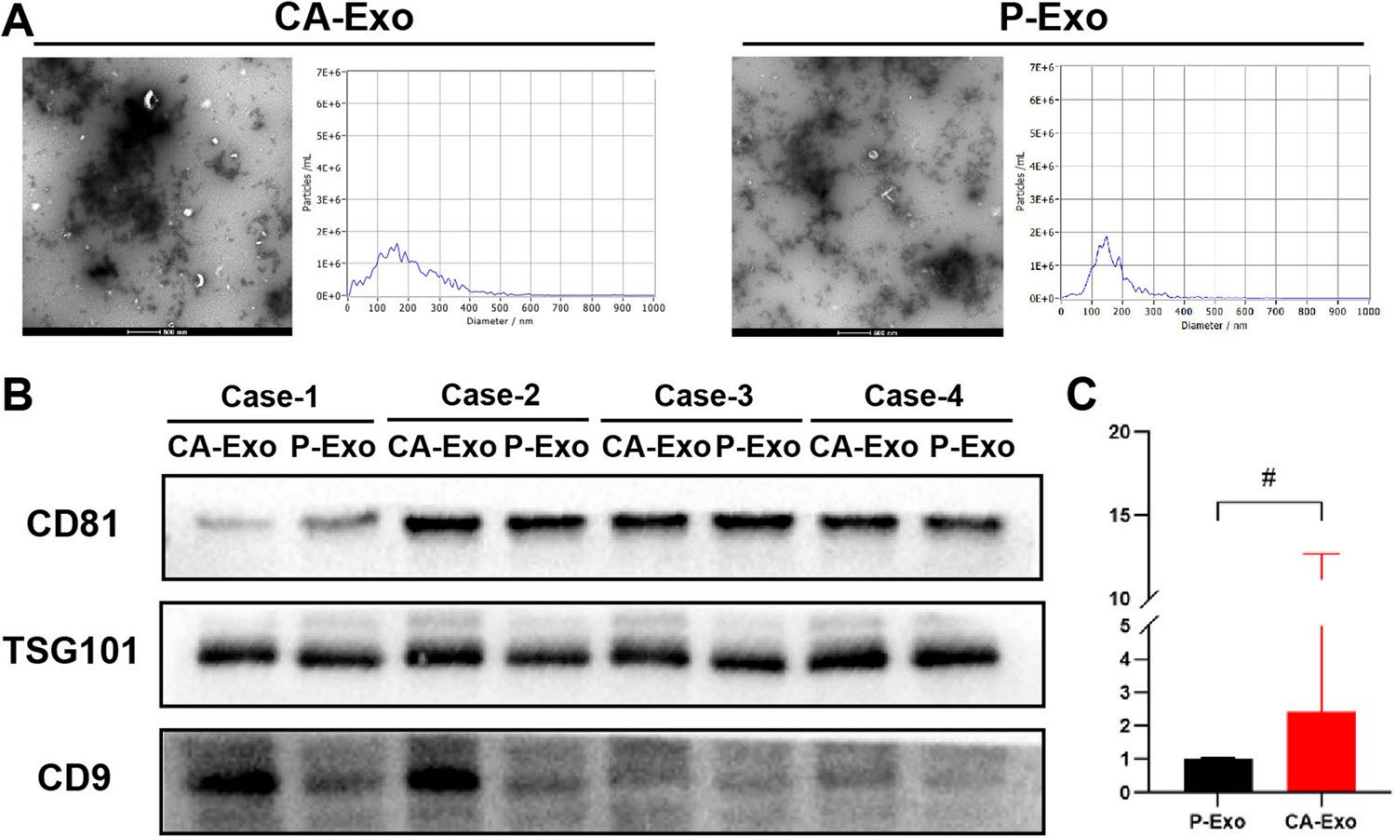
Differential expression analysis of miR-3529-3p in clinical samples. **A**, **B** Biological characterization of exosomes extracted from cancer and paracancerous tissues; **C** statistics of differential expression of exosomal miR-3529-3p in cancer and paracancerous tissues. ^#^*P* < 0.05

The above three miRNAs were validated in CAF-Exos and hOMF-Exos by qRT‒PCR. The results showed that miR-3529-3p and miR-3074-5p were higher in CAF-Exos than in hOMF-Exos (Fig. 5B). After treatment of Cal-27 cells with hOMF-Exos and CAF-Exos for 48 h, the expression of all three miRs was significantly higher in Cal-27 cells in the CAF-Exo group than in the hOMF-Exo group (Fig. 5C). MiR-3529-3p expression was significantly elevated in hOMF cells after treatment with PDGF-BB-containing medium (30 ng/mL PDGF-BB, serum-free, high glucose DMEM) for 72 h compared to that of untreated hOMFs (*P* = 0.0020). MiR-374c-3p and miR-3074-5p expression was also upregulated but with no statistical significance (Fig. 5D).

### MiR-3529-3p is differentially expressed in samples from patients with oral squamous carcinoma

A total of 26 tissue samples from OSCC patients were collected in this study. Twenty patients (76.92%) were male, five patients (19.23%) were female, and one patient (3.84%) had missing information. The mean age was 55.16 ± 11.68 years, with an age range of 25–73 years and a median age of 56 years. The mean height was 163.88 ± 6.69 cm, with a range of 151–182 cm. The mean weight was 62.04 ± 12.24 kg, with a range of 42–95 kg. The mean BMI of the patients was 23.06 ± 3.88, with a minimum BMI of 16.82 and a maximum of 31.41 (Supplementary Table 5).

In this study, exosomes were successfully extracted from 20 clinical samples of cancer and adjacent tissues. Transmission electron microscopy results showed that the exosomes were approximately 100 nm in size and had a bilayer-like tea tray structure. The NTA nanoparticle size results showed that the particle size of exosomes was distributed at approximately 100 nm, and the particle concentration was approximately 10 E^10^/mL. The expression of CD9, CD81, and TSG101 in exosomes, which are the main surface markers, was examined by WB (Fig. 5A, B). The expression of miR-3529-3p was detected in exosomes by qRT‒PCR, and it was significantly higher in exosomes from cancerous tissues than in those from adjacent tissues (Supplementary Table 6, Fig. 5C).

### MiR-3529-3p promotes proliferation, migration, invasion, and antiapoptosis in Cal-27 cells

**Fig. 6:**
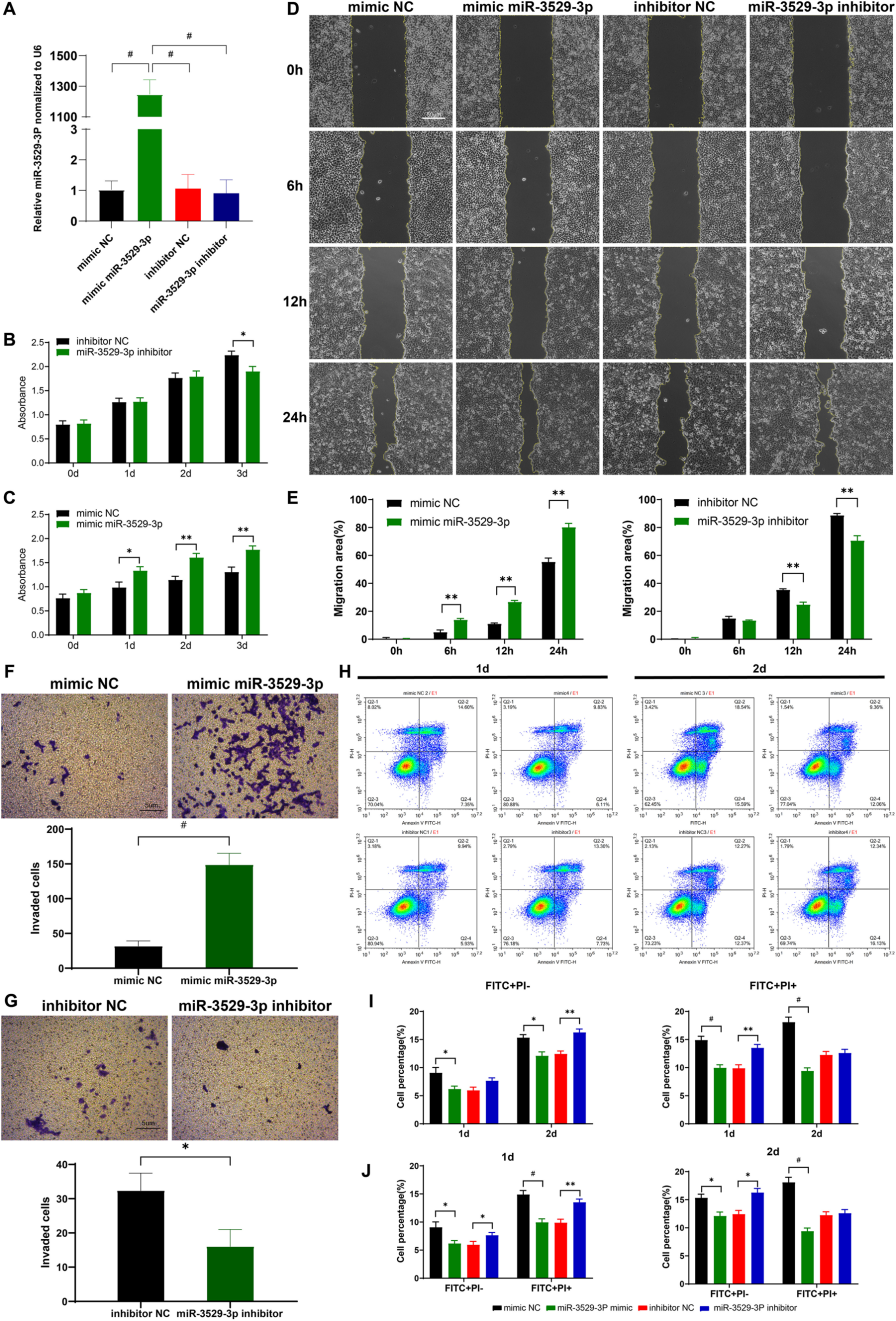
The Effect of miR-3529-3p on the proliferation, migration, invasion, and anti-apoptotic ability of Cal-27. **A** Expression level of miR-3529-3p by qRT-PCR. **B** Cell proliferation in mimic NC and mimic 3529 groups. **C** Proliferation in inhibit NC and miR-3529-3p inhibitor groups. **D**, **E** Cal-27 cell migration during 0-24 h of miR-3529-3p transfection and its quantitative analysis. **F**, **G** Transwell invasive assay results of four cell lines and their statistical analysis. **H** Apoptosis assay of four cell lines. **I** Statistical analysis of early apoptosis (FITC+ PI−) and late apoptosis (FITC+ PI+). **J** Statistical analysis of flow cytometric results

Cal-27 cells were transfected with miR-3529-3p mimic/inhibitor, mimic NC, and inhibitor NC at 100 nM. qRT‒PCR results showed that the relative expression of miR-3529-3P mimic was significantly higher in Cal-27 cells (1246.89-fold) compared to that of the negative control mimics NC after 48 h transfection. There was no significant change in the relative expression of miR-3529-3p in Cal-27 cells transfected with miR-3529-3p inhibitor compared to the negative control inhibitor NC (Fig. 6A).

The results of the CCK8 assay showed that miR-3529-3p overexpression promoted the proliferation of Cal-27 cells at 1, 2, and 3 days compared to that of the mimic NC group (P < 0.05). MiR-3529-3p knockdown did not significantly inhibit the proliferation of Cal-27 cells on Days 1 and 2 but significantly inhibited Cal-27 cell proliferation on the 3rd day (Fig. 6B, C). Wound healing assay results showed that miR-3529-3p overexpression promoted cell migration (6 h, P = 0.0073; 12 h, P = 0.0001; 24 h, P = 0.0027). MiR-3529-3p knockdown inhibited cell migration (12 h, P = 0.004; 24 h, P = 0.0077) (Fig. 6D, E). Transwell invasion assays showed that miR-3529-3p overexpression promoted cell invasion (P = 0.0004) and that knockdown of miR-3529-3p inhibited cell invasion (P = 0.0168) (Fig. 6F, G).

The miR-3529-3p mimic group showed a decrease in both early (FITC+PI−) and late (FITC+PI+) apoptosis in Cal-27 cells compared with the mimic NC group, with a more significant effect on the proportion of late apoptotic cells. The miR-3529-3p inhibitor group showed an increase in both early and late apoptosis in Cal-27 cells compared to the inhibitor NC group, with a more significant effect on increasing the proportion of early apoptotic cells. The miR-3529-3p mimic reduced the proportion of late apoptosis in Cal-27 cells throughout the transfection time course, whereas the miR-3529-3p inhibitor increased the proportion of early apoptosis throughout the transfection period, but the effect of the miR-3529-3p inhibitor on promoting late apoptosis was observed only on Day 1 after transfection (Fig. 6H–J). The results indicated that miR-3529-3p overexpression significantly enhanced the antiapoptotic ability of Cal-27 cells.

### MiR-3529-3p regulates malignant behavior and patient prognosis of OSCC through multiple potential target genes

**Fig. 7:**
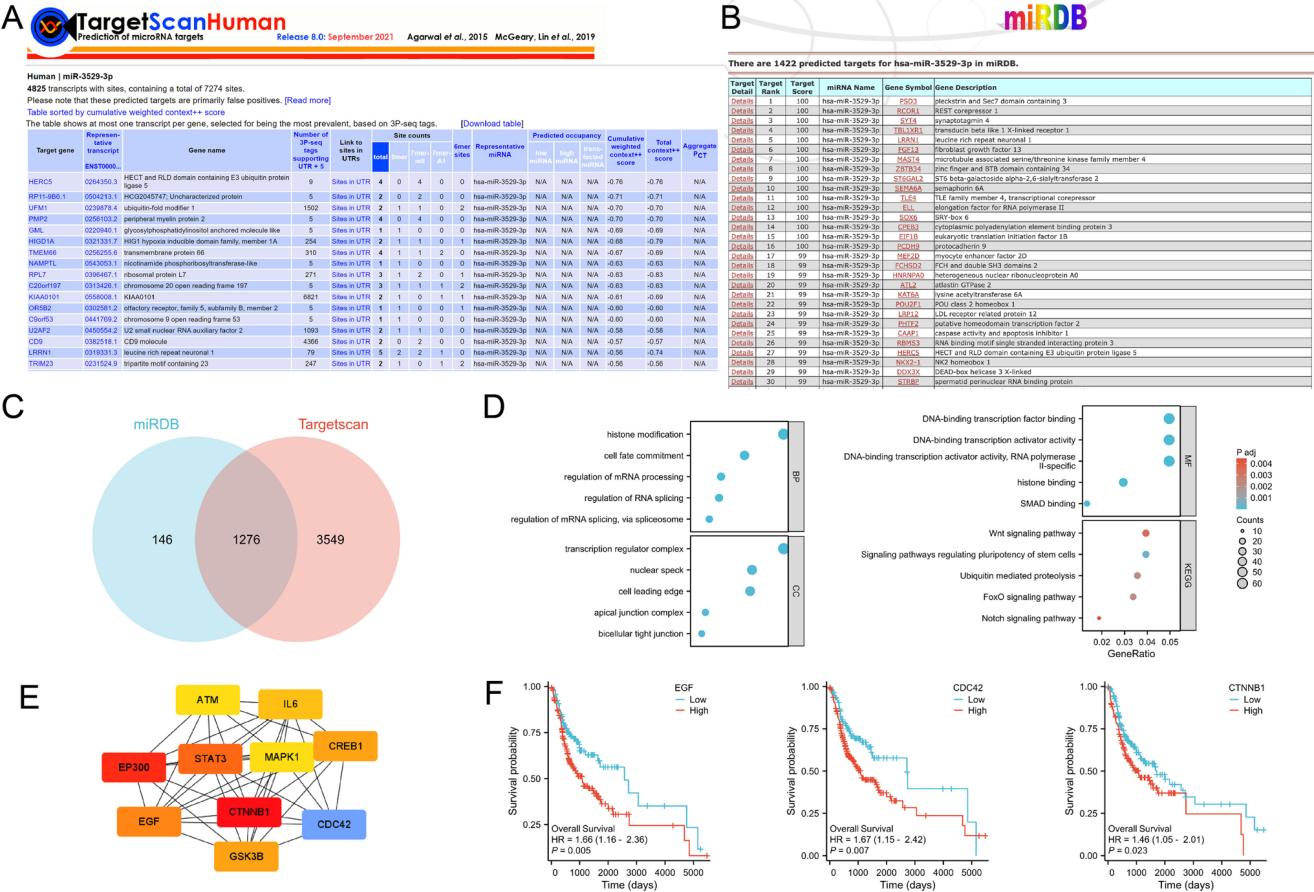
Target gene prediction of MiR-3529-3p and its functional analysis. **A** TargetScanHuman database predicts the target genes of miR-3529-3p. **B** Prediction of miR-3529-3p target genes by miRDB database. **C** Venn diagram of two sites predicting target genes. **D** Bubble map of GO/KEGG analysis of 1276 potential target genes. **E** PPI network diagram of hub genes among 1276 potential target genes (Red: high correlation; Blue: low correlation). **F** Prognosis-related KM curves of EGF, CDC42 and CTNNB1 in OSCC

We predicted the target genes of miR-3529-3p through the TargetScanHuman database and miRDB database (Fig. 7A-B, Supplementary Table 7). We further intersected the predicted target genes from the two sites and obtained a total of 1276 potential target genes (Fig. 7C). Then, we subjected the obtained potential target genes to GO and KEGG enrichment analysis (Fig. 7D). KEGG pathway enrichment was mainly associated with Wnt signaling pathway, Signaling pathways regulating pluripotency of stem cells, Ubiquitin mediated proteolysis, FoxO signaling pathway, and Notch signaling pathway, which we speculate may be an important signaling pathway for miR-3529-3p to mediate oral squamous cancer progression through target genes. Finally, we made a PPI network map of 1276 potential target genes and looked for hub genes (Fig. 7E), three of which had a positive impact on the survival prognosis of oral squamous carcinoma patients (Fig. 7F).

## Discussion

OSCC is a malignant tumour originating from the oral epithelium. Surgical resection combined with radiotherapy is currently the main treatment for OSCC, but conventional treatment has a poor prognosis and many side effects. It is important to investigate the potential molecular mechanisms and new therapeutic targets of OSCC. The tumour microenvironment has an important role in regulating the development of OSCC, and the most abundant cells are CAFs. Activated CAFs undergo major changes in morphological structure, growth pattern, proliferative activity, motility, and secretory function. These cells can regulate the tumour microecological system through direct cell‒cell contact, secretion of soluble factors, and modification of the extracellular mesenchyme to promote tumorigenesis, progression, and metastasis [13]. It is important to investigate how CAFs contribute to the malignancy of OSCC.

CAFs were first isolated from prostate cancer tissue by Olumi et al. in 1999 and were shown to promote tumour evolution [14]. CAFs are generally derived from host quiescent fibroblasts induced by a variety of cytokines secreted by tumour cells (e.g., TGF-β, PDGF-BB, and fibroblast growth factor) [13, 15]. When stimulated by external factors, resting fibroblasts are activated into CAFs, which vary in shape and function from tissue to tissue. PDGF-BB and its receptors promote tumorigenesis and progression and are closely associated with CAFs [16]. The PDGF family plays a key role in embryonic development, cell growth, cell differentiation, and the tissue damage response [17, 18]. Aoto et al. [19] and Rizviet et al. [20] found that PDGF-BB plays an important role in supporting the formation of CAFs in the tumour microenvironment. Zhang et al. [21] demonstrated that PDGF-BB can induce the conversion of human normal fibroblasts into CAFs. Ren et al. [9] found that PDGF-BB can regulate the conversion of fibroblasts to CAFs via the lncRNA LURAP1L-AS1/LURAP1L/IKK/IκB/NF-κB signalling pathway. Our study likewise revealed that hOMF activation of CAFs was induced using PDGF-BB at a concentration of 30 ng/mL.

Exosomes secreted by different cells, such as tumour cells, stromal cells, and immune cells, play different roles and are involved in the formation of the tumour microenvironment and tumour progression [22]. Exosomes play an important role in promoting chemoresistance, antiapoptosis, DNA damage repair regulation, and immunomodulation in OSCC through signalling transmission between tumour cells [23]. The composition and function of exosomes were also associated with tumour susceptibility genes (TSG101), apoptosis-related gene 2-interacting protein X, endosomal sorting complexes required for transporter proteins, and soluble *N*-ethylmaleimide-sensitive factor attachment protein receptor complex proteins [24, 25]. Moreover, exosomes released from cells can interact with target cells in three ways. First, there is fusion of the plasma membrane between the target cells and the exosomes. The second is the binding of the target cell membrane receptor to proteins on the surface of the exosome vesicles. Finally, exosomes can also be transformed in vivo by endocytosis of the target cells [26]. Our transmission electron microscopy results showed that both hOMF-Exos and CAF-Exos had a typical circular bilayer-like structure, and the peak particle size followed the relevant literature [27]. Coculture of PKH26-labelled hOMF-Exos/CAF-Exos with OSCC cells (Cal-27, HSC-4, SCC-6) revealed that OSCC cells can engulf exosomes. In this study, we further compared the effects of hOMF-Exos/CAF-Exos on the proliferation, migration, invasion, and antiapoptosis of Cal-27 cells. Compared with hOMF-Exos, CAF-Exos had significantly stronger pro-proliferative, migratory, and invasive effects and superior antiapoptotic effects on Cal-27 cells. The Transwell coculture model also confirmed the above findings. In vivo, tumorigenic assays showed that the total weight and volume of tumours in the CAF-Exo group were significantly greater than those in the NC and hOMF-Exo groups. These results suggest that CAF-Exos can promote tumour growth by exerting antiapoptotic and pro-proliferative effects. This finding is generally consistent with the literature [28]. Bhagyashri [29] also found that miR-30a in exosomes suppressed tumours by inhibiting apoptosis in OSCC.

This study also found that the differences in proliferation, migration, invasion, and antiapoptotic effects of hOMF-Exos and CAF-Exos on Cal-27 cells were less than those of the hOMF-Exo/CAF-Exo and NC groups. The possible reasons are as follows: the function of exosomes is associated with their concentration [27]. In our experiments, the activation of hOMFs into CAFs resulted in a more vigorous secretion of exosomes. Not only the difference in the exosome content itself but also the difference in the number of exosomes secreted affects the biology of the cells. Therefore, the same concentration of treatment does not fully mimic the real CAF-Exo function in vivo. This possibility is also suggested by the difference in experimental results between the transwell coculture model and the direct extraction of exosomes in this section. However, limited by human and material resources and technical means, we have not yet validated this issue. In addition, CAFs, as one of the most important components of the tumour microenvironment, exhibit biological heterogeneity and functional differences in several aspects [30]. However, the subtype of CAFs derived from PDGF-BB-induced hOMF activation has not yet been validated due to limited human and material resources.

Exosomes contain a variety of biologically active molecules, such as proteins, lipids, and nucleic acids. Of these, miRNAs play an important role in cellular transport and signal transduction [31, 32]. Exosomes can communicate among cells through miRNA exchange and thus contribute to tumour development [33–35]. The previous understanding was that exosomes from the same type of cell contain similar proteins, nucleic acids, and lipids and perform similar functions. However, recent studies have shown that the contents of exosomes depend not only on the cell type but also on the origin of the cells [36, 37]. Proteomic analysis of breast cancer cells and their exosomes revealed that the exosomes secreted by epithelial and mesenchymal cells contained different proteins and nucleic acids. Moreover, exosomes secreted by cancer cells and noncancerous cells contain different cholesterol and phospholipids [38, 39].

CAF-derived miRNAs were found to enhance the metastatic ability of OSCC by activating multiple signalling pathways. For example, miR-382-5p in CAF-Exos can promote the migration of tumour cells in OSCC [12]. Expression of miR-3188 is reduced in exosomes from head and neck cancer tissues and their parental CAFs. Downregulation of miR-3188 promotes the expression of its direct target, Bcl-2, which promotes the transition of the cell cycle from G1 to S and colony formation capacity and reduces apoptosis [40]. Previous studies found that miR-34a-5p is significantly reduced in CAF-Exos, which directly upregulates AXL and further promotes tumour progression by increasing the activation of SNAIL [39]. In OSCC, CAFs are derived from hOMF activation, which may have altered the composition of secreted exosomes and thus influenced tumour progression. In this study, a total of 39 differentially expressed miRNAs in CAF-Exos and hOMF-Exos were screened. The results of gene functional enrichment for the top 5 miRNAs showed that miRNA target genes were mainly enriched in immune and tumour-associated pathways. The results demonstrate the heterogeneity of CAF-Exos and a potential regulatory role for OSCC.

MiR-3529-3p had a significant effect on Cal-27 cells. Knockdown of miR-3529-3p significantly inhibited the proliferation, migration, and invasion of Cal-27 cells. Its overexpression promoted cell proliferation, migration, and invasion, while the miR-3529-3p inhibitor increased the rate of apoptotic cell death. The above findings confirmed that miR-3529-3p significantly promoted the proliferative and antiapoptotic effects of OSCC, which was generally consistent with the findings of related studies. Studies on the promotion of OSCC malignancy by miR-3529-3p in exosomes have not been reported. However, miR-3529-3p was found to have a tumour-regulating effect in a related study. The results of Weng et al. [41] confirmed that inhibition of miR-3529-3p significantly reduces the migration and invasion of hepatocellular carcinoma cells, suggesting that miR-3529-3p is an important regulator of tumours. Kinget et al. [42] found that miR-3529-3p is negatively correlated with VEGFR1/VEGFR2 expression. This molecule correlated with shrinkage and progression-free survival after VEGFR-TKI treatment in metastatic clear cell renal cell carcinoma and has predictive value for oncologic therapy evaluation.

CAFs in different tumor tissues can produce and secrete different levels of miRNAs. Studies have shown that CAF-derived miRNAs, both up- and downregulated, can have a dramatic impact on the self-activation, tumorigenesis, metastasis, angiogenesis, immunosuppression, and chemoresistance of CAFs [43]. Moreover, CAF-derived miRNAs can weaken the repression of relevant oncogenes. This process promotes tumorigenesis and malignant disease progression through oncogene activation, cell cycle regulation, and reduction of apoptosis [44]. In this study, we found that the key molecule of exosomes promoting the malignant behavior of OSCC is miR-3529-3p, and multiple potential target genes of miR-3529-3p may affect the malignant behavior of OSCC and patient survival through multiple pathways. Wnt signaling pathway has been documented to promote proliferation, migration, and invasion of oral squamous carcinoma cells [45, 46]. Notch signaling pathway also promotes proliferation, and invasion and affects the patient prognosis of oral squamous carcinoma cells [47, 48]. Inhibition of the FoxO signaling pathway has also been shown to effectively inhibit the proliferation of oral squamous carcinoma cells [49]. However, miR-3529-3p interacts with potential target genes to mediate the above signaling pathway to regulate OSCC progression has not been reported, which is the focus of our next study. In addition, the prognostic value of miR-3529-3p and OSCC could not be successfully determined, mainly because there were no data on exosomal miR-3529-3p in TCGA and GEO data, and the correlation data for head and neck squamous cancer tissue expression of miR-3529-3p were missing up to more than 95%. In future work, clinical cohorts need to be established to analyse the diagnostic and prognostic efficacy of miR-3529-3p.

## Conclusion

**Fig. 8:**
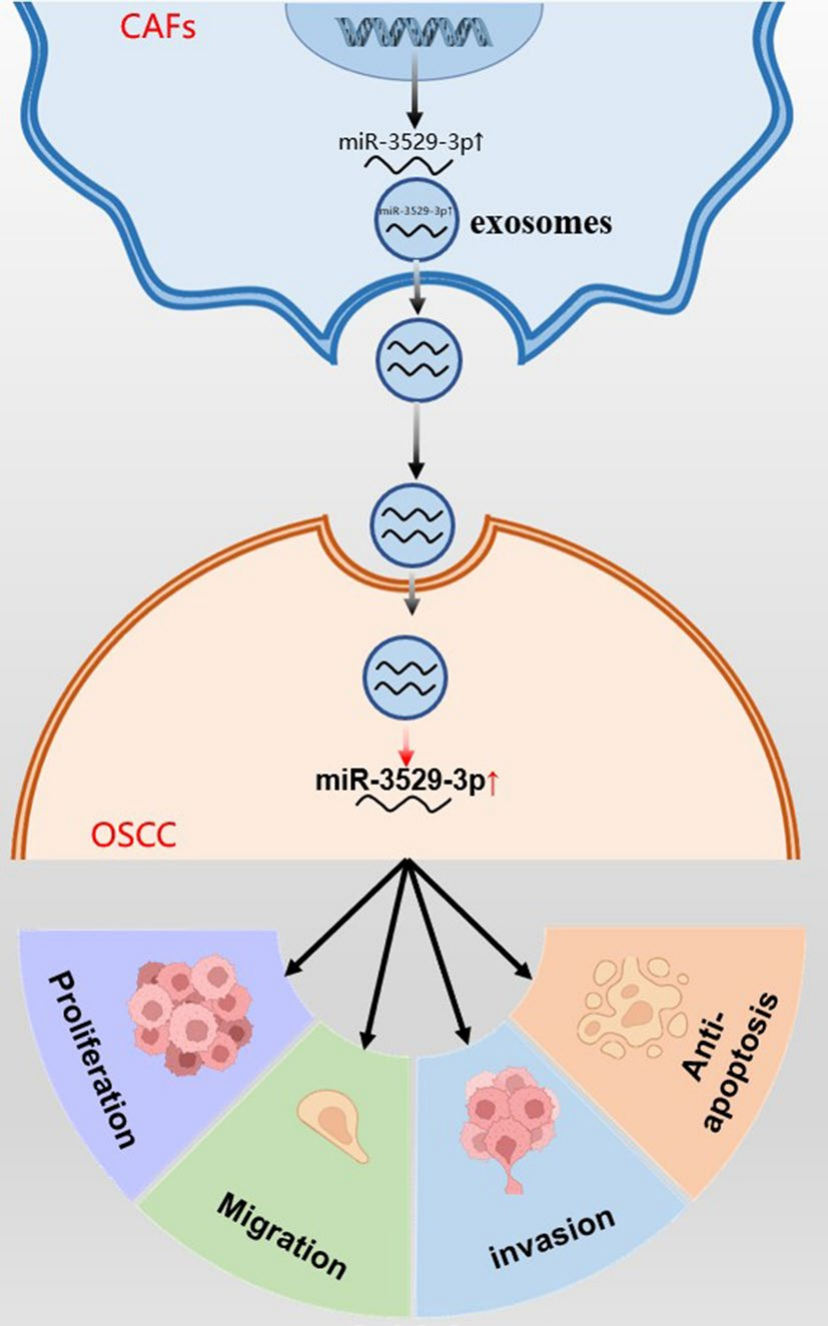
Mechanism of CAFs-Exo promoting malignant behavior in oral squamous cell carcinoma

PDGF-BB induces the activation of hOMFs into CAFs, and its secreted exosomes promote the proliferation, migration, and invasion of Cal-27 cells and significantly enhance their antiapoptotic ability. CAF-Exos promote tumour growth and migration by exerting antiapoptotic and proproliferative effects. MiR-3529-3p is abundant in CAF-Exos and is significantly higher than that in hOMF-Exos. MiR-3529-3p effectively promoted the proliferation, migration, and invasion of Cal-27 cells and significantly enhanced the antiapoptotic ability of Cal-27 cells (Fig. 8). In conclusion, miR-3529-3p, which is enriched in CAF-Exos, is a key molecule that plays a role in promoting the malignant behaviour of OSCC, and HERC5 is a potential key regulatory target gene of miR-3529-3p.

### Supplementary Information

Below is the link to the electronic supplementary material.

Supplementary file1 (DOCX 21 KB)

Supplementary file2 (DOCX 15 KB)

Supplementary file3 (DOCX 29 KB)

Supplementary file4 (DOCX 28 KB)

Supplementary file5 (DOCX 26 KB)

Supplementary file6 (DOCX 24 KB)

Supplementary file7 (XLSX 76 KB)

Supplementary file8 (DOCX 508 KB)

Supplementary file9 (TIF 144873 KB)

## Data Availability

The datasets supporting the conclusions of this article are available by contacting the corresponding author (Email: bianli@kmmu.edu.cn). The Supplementary Material for this article can be found online. All sequencing data have been uploaded to the GEO database with the accession number Series GSE222278.

## References

[1] Bray F, Ferlay J, Soerjomataram I (2018). Global cancer statistics 2018: GLOBOCAN estimates of incidence and mortality worldwide for 36 cancers in 185 countries. CA Cancer J Clin.

[2] Lebleu VS, Kalluri R (2020). Exosomes as a multicomponent biomarker platform in cancer. Trends Cancer.

[3] Ng JH, Iyer NG, Tan MH (2017). Changing epidemiology of oral squamous cell carcinoma of the tongue: a global study. Head Neck.

[4] Maas SLN, Breakefield XO, Weaver AM (2017). Extracellular vesicles: unique intercellular delivery vehicles. Trends Cell Biol.

[5] Mao X, Xu J, Wang W (2021). Crosstalk between cancer-associated fibroblasts and immune cells in the tumor microenvironment: new findings and future perspectives. Mol Cancer.

[6] Chen F, Zhuang X, Lin L (2015). New horizons in tumor microenvironment biology: challenges and opportunities. BMC Med.

[7] Peltanova B, Raudenska M, Masarik M (2019). Effect of tumor microenvironment on pathogenesis of the head and neck squamous cell carcinoma: a systematic review. Mol Cancer.

[8] Li Z, Liu J, Li L (2018). Epithelial mesenchymal transition induced by the CXCL9/CXCR3 axis through AKT activation promotes invasion and metastasis in tongue squamous cell carcinoma. Oncol Rep.

[9] Ren X, Li L, Wu J (2021). PDGF-BB regulates the transformation of fibroblasts into cancer-associated fibroblasts via the lncRNA LURAP1L-AS1/LURAP1L/IKK/IκB/NF-κB signaling pathway. Oncol Lett.

[10] Tassinari M, Gandellini P (2021). Noncoding RNAs in the interplay between tumor cells and cancer-associated fibroblasts: signals to catch and targets to hit. Cancers.

[11] Yang F, Ning Z, Ma L (2017). Exosomal miRNAs and miRNA dysregulation in cancer-associated fibroblasts. Mol Cancer.

[12] Sun LP, Xu K, Cui J (2019). Cancer-associated fibroblast-derived exosomal miR-382-5p promotes the migration and invasion of oral squamous cell carcinoma. Oncol Rep.

[13] Kumar D, New J, Vishwakarma V (2018). Cancer-associated fibroblasts drive glycolysis in a targetable signaling loop implicated in head and neck squamous cell carcinoma progression. Cancer Res.

[14] Olumi AF, Grossfeld GD, Hayward SW (1999). Carcinoma-associated fibroblasts direct tumor progression of initiated human prostatic epithelium. Cancer Res.

[15] Liu J, Ren L, Li S (2021). The biology, function, and applications of exosomes in cancer. Acta Pharm Sin B.

[16] Gialeli C, Nikitovic D, Kletsas D (2014). PDGF/PDGFR signaling and targeting in cancer growth and progression: focus on tumor microenvironment and cancer-associated fibroblasts. Curr Pharm Des.

[17] Huber L, Birk R, Rotter N (2020). Effect of small-molecule tyrosine kinase inhibitors on PDGF-AA/BB and PDGFRα/β expression in SCC according to HPV16 status. Anticancer Res.

[18] Ouyang L, Zhang K, Chen J (2018). Roles of platelet-derived growth factor in vascular calcification. J Cell Physiol.

[19] Aoto K, Ito K, Aoki S (2018). Complex formation between platelet-derived growth factor receptor β and transforming growth factor β receptor regulates the differentiation of mesenchymal stem cells into cancer-associated fibroblasts. Oncotarget.

[20] Rizvi S, Mertens JC, Bronk SF (2014). Platelet-derived growth factor primes cancer-associated fibroblasts for apoptosis. J Biol Chem.

[21] Zhang D, Wang Y, Shi Z (2015). Metabolic reprogramming of cancer-associated fibroblasts by IDH3α downregulation. Cell Rep.

[22] Yang WW, Yang LQ, Zhao F (2017). Epiregulin promotes lung metastasis of salivary adenoid cystic carcinoma. Theranostics.

[23] Law ZJ, Khoo XH, Lim PT (2021). Extracellular vesicle-mediated chemoresistance in oral squamous cell carcinoma. Front Mol Biosci.

[24] Bebelman MP, Smit MJ, Pegtel DM (2018). Biogenesis and function of extracellular vesicles in cancer. Pharmacol Ther.

[25] Ciardiello C, Cavallini L, Spinelli C (2016). Focus on extracellular vesicles: new frontiers of cell-to-cell communication in cancer. Int J Mol Sci.

[26] Żbikowski A, Błachnio-Zabielska A, Galli M (2021). Adipose-derived exosomes as possible players in the development of insulin resistance. Int J Mol Sci.

[27] Kalluri R, Lebleu VS (2020). The biology, function, and biomedical applications of exosomes. Science.

[28] Zhao C, Zhang G, Liu J (2020). Exosomal cargoes in OSCC: current findings and potential functions. PeerJ.

[29] Kulkarni B, Gondaliya P, Kirave P (2020). Exosome-mediated delivery of miR-30a sensitize cisplatin-resistant variant of oral squamous carcinoma cells via modulating Beclin1 and Bcl2. Oncotarget.

[30] Liu T, Han C, Wang S (2019). Cancer-associated fibroblasts: an emerging target of anti-cancer immunotherapy. J Hematol Oncol.

[31] Castellanos-Rizaldos E, Grimm DG, Tadigotla V (2018). Exosome-based detection of EGFR T790M in plasma from non-small cell lung cancer patients. Clin Cancer Res.

[32] Zhang Z, Xing T, Chen Y (2018). Exosome-mediated miR-200b promotes colorectal cancer proliferation upon TGF-β1 exposure. Biomed Pharmacother.

[33] Whiteside TL (2018). Exosome and mesenchymal stem cell cross-talk in the tumor microenvironment. Semin Immunol.

[34] Wu CX, Liu ZF (2018). Proteomic profiling of sweat exosome suggests its involvement in skin immunity. J Invest Dermatol.

[35] Guay C, Regazzi R (2017). Exosomes as new players in metabolic organ cross-talk. Diabetes Obes Metab.

[36] Bang C, Thum T (2012). Exosomes: new players in cell-cell communication. Int J Biochem Cell Biol.

[37] Jan AT, Rahman S, Badierah R (2021). Expedition into exosome biology: a perspective of progress from discovery to therapeutic development. Cancers (Basel).

[38] Luga V, Wrana JL (2013). Tumor-stroma interaction: revealing fibroblast-secreted exosomes as potent regulators of Wnt-planar cell polarity signaling in cancer metastasis. Cancer Res.

[39] Li YY, Tao YW, Gao S (2018). Cancer-associated fibroblasts contribute to oral cancer cells proliferation and metastasis via exosome-mediated paracrine miR-34a-5p. EBioMedicine.

[40] Wang X, Qin X, Yan M (2019). Loss of exosomal miR-3188 in cancer-associated fibroblasts contributes to HNC progression. J Exp Clin Cancer Res.

[41] Weng Z, Peng J, Wu W (2021). Downregulation of PART1 inhibits proliferation and differentiation of Hep3B cells by targeting hsa-miR-3529-3p/FOXC2 Axis. J Oncol.

[42] Kinget L, Roussel E, Verbiest A (2021). MicroRNAs targeting HIF-2α, VEGFR1 and/or VEGFR2 as potential predictive biomarkers for VEGFR tyrosine kinase and HIF-2α inhibitors in metastatic clear-cell renal cell carcinoma. Cancers (Basel).

[43] Monteran L, Erez N (1835). The dark side of fibroblasts: cancer-associated fibroblasts as mediators of immunosuppression in the tumor microenvironment. Front Immunol.

[44] Wang X, Wang X, Xu M (2021). Effects of CAF-derived microRNA on tumor biology and clinical applications. Cancers (Basel).

[45] Dai BW, Yang ZM, Deng P (2019). HOXC10 promotes migration and invasion via the WNT-EMT signaling pathway in oral squamous cell carcinoma. J Cancer.

[46] Zheng TL, Cen K (2020). MiR-92a inhibits proliferation and promotes apoptosis of OSCC cells through Wnt/β-catenin signaling pathway. Eur Rev Med Pharmacol Sci.

[47] Guo Y, Chen Y, Liu H (2020). Alpinetin inhibits oral squamous cell carcinoma proliferation via miR-211-5p upregulation and notch pathway deactivation. Nutr Cancer.

[48] Weaver AN, Burch MB, Cooper TS (2016). Notch signaling activation is associated with patient mortality and increased FGF1-mediated invasion in squamous cell carcinoma of the oral cavity. Mol Cancer Res.

[49] Fang L, Wang H, Zhou L (2011). Akt-FOXO3a signaling axis dysregulation in human oral squamous cell carcinoma and potent efficacy of FOXO3a-targeted gene therapy. Oral Oncol.

