# Supplementary material for: MiR-3529-3p from PDGF-BB-induced cancer-associated fibroblast-derived exosomes promotes the malignancy of oral squamous cell carcinoma
Source: Discov Oncol. 2023 Sep 5;14:166. doi: 10.1007/s12672-023-00753-9 (PMC10480386; doi:10.1007/s12672-023-00753-9)
Supplement: Supplementary file 1 — Supplementary file1 (DOCX 21 KB) [file 12672_2023_753_MOESM1_ESM.docx]

Supplementary table 1. mRNA primer list

| Gene | | Forward primer (5'-3') | Reverse primer (5'-3') | |  |
| --- | --- | --- | --- | --- | --- |
| α-SMA | | AGCGTGGCTATTCCTTCGTT | GCCCATCAGGCAACTCGTAA | |  |
| FAP | | TTATGCTGGTCGCCTGTTGG | AGGAGACCACCAGAGAGCATA | |  |
| β-tublin | | TCTCCTTATCAGCAAGATCCGAG | TTCAGATCCCCGTAGGTTGGT | |  |
| HERC5 | | CTCTTGCACCGTCTCAATTTTT | GGAAAGTGACTGAATATGACGC | |  |
| GAPDH | GAAGGTCGGAGTCAACGGATTT | | | GCCATGGGTGGAATCATATTGG | |

Supplementary table 1-1. miRNA primer table

| Gene | Sequence（5^，^→3 ^,^） |
| --- | --- |
| miR-3529-3p Forward primer | GCTTGGCTCGTGGAAGAAGGAG |
| mRQ 3’Primer | Reagent kit provided |
| U6 Forward primer | Reagent kit provided |
| U6 Reverse primer | Reagent kit provided |
