# Supplementary material for: MiR-3529-3p from PDGF-BB-induced cancer-associated fibroblast-derived exosomes promotes the malignancy of oral squamous cell carcinoma
Source: Discov Oncol. 2023 Sep 5;14:166. doi: 10.1007/s12672-023-00753-9 (PMC10480386; doi:10.1007/s12672-023-00753-9)
Supplement: Supplementary file 2 — Supplementary file2 (DOCX 15 KB) [file 12672_2023_753_MOESM2_ESM.docx]

Supplementary table 2. Antibody information table

| Antibody name | Operating concentration |
| --- | --- |
| FAP | 1:1000 |
| α-SMA | 1:5000 |
| β-tubulin | 1:5000 |
| CD9 | 1:1000 |
| CD63 | 1:1000 |
| CD81 | 1:1000 |
