# Supplementary material for: MiR-3529-3p from PDGF-BB-induced cancer-associated fibroblast-derived exosomes promotes the malignancy of oral squamous cell carcinoma
Source: Discov Oncol. 2023 Sep 5;14:166. doi: 10.1007/s12672-023-00753-9 (PMC10480386; doi:10.1007/s12672-023-00753-9)
Supplement: Supplementary file 3 — Supplementary file3 (DOCX 29 KB) [file 12672_2023_753_MOESM3_ESM.docx]

**Supplementary table 3. Statistics of differential miRNAs in CAFs-Exo and hOMF-Exo**

| **差异miRNA** | | **CAFs-Exo** | | | | ***X±SD*** |  | **hOMF-Exo** | | | ***‾X±SD*** | ***P*** | ***log2 (CAFs / hOMF)*** |  |  |  |
| --- | --- | --- | --- | --- | --- | --- | --- | --- | --- | --- | --- | --- | --- | --- | --- | --- |
|  |  | **CAFs1** | **CAFs2** | | **CAFs3** |  |  | **hOMF1** | **hOMF2** | **hOMF3** |  |  |  |  |  |  |
| **hsa-miR-3529-3p** | | | **860.13** | | | **257.07** | **1.60** | **372.93±440.83** |  | **414.63** | **0.96** | **0.46** | **138.68±238.98** | **＜0.001** | **1.43** | |
| **hsa-miR-3074-5p** | | | **166.11** | | | **150.64** | **298.49** | **205.08±81.26** |  | **74.77** | **1152.74** | **121.67** | **449.73±609.28** | **＜0.001** | **-1.13** | |
| **hsa-miR-92b-3p** | | | **13.08** | | | **23.23** | **24.41** | **20.24±6.23** |  | **29.50** | **34.37** | **68.24** | **44.04±21.10** | **＜0.001** | **-1.12** | |
| **hsa-miR-374c-3p** | | | **11.05** | | | **11.62** | **11.41** | **11.36±0.29** |  | **0** | **4.48** | **11.80** | **5.42±5.96** | **＜0.001** | **1.07** | |
| **hsa-miR-96-5p** | | | **6.85** | | | **7.68** | **8.20** | **7.58±0.68** |  | **3.89** | **37.09** | **6.48** | **15.82±18.47** | **＜0.001** | **-1.062** | |
| novel-hsa-miR104-5p | | 4.36 | | | 4.50 | 11.94 | 6.93±4.34 |  | 3.89 | 1.76 | 0.93 | 2.19±1.53 | ＜0.001 | 1.66 | |  |
| novel-hsa-miR294-3p | | 8.25 | | | 7.12 | 0.36 | 5.24±4.270 |  | 0.41 | 0.48 | 0.23 | 0.37±0.13 | ＜0.001 | 3.81 | |  |
| hsa-miR-30c-5p | | 4.20 | | | 5.62 | 5.88 | 5.24±0.90 |  | 12.50 | 6.55 | 14.80 | 11.29±4.26 | ＜0.001 | -1.11 | |  |
| novel-hsa-miR237-3p | | 7.94 | | | 1.12 | 4.63 | 4.566±3.41 |  | 2.05 | 2.24 | 1.39 | 1.89±0.45 | ＜0.001 | 1.27 | |  |
| novel-hsa-miR293-5p | | 2.34 | | | 6.75 | 4.46 | 4.51±2.21 |  | 7.38 | 7.19 | 14.11 | 9.56±3.94 | ＜0.001 | -1.08 | |  |
| hsa-miR-4488 | | 3.74 | | | 2.06 | 4.10 | 3.30±1.09 |  | 0.21 | 1.28 | 1.62 | 1.03±0.74 | ＜0.001 | 1.67 | |  |
| hsa-let-7g-3p | | 2.65 | | | 1.69 | 4.81 | 3.05±1.60 |  | 1.64 | 1.76 | 0.93 | 1.44±0.45 | ＜0.001 | 1.08 | |  |
| novel-hsa-miR306-3p | | | 1.87 | | 2.99 | 4.10 | 2.99±1.12 |  | 14.34 | 13.59 | 1.62 | 9.85±7.14 | ＜0.001 | -1.7 | |  |
| novel-hsa-miR283-5p | | | 1.25 | | 3.19 | 4.46 | 2.96±1.62 |  | 0 | 3.36 | 0.23 | 1.20±1.88 | ＜0.001 | 1.31 | |  |
| novel-hsa-miR35-5p | | | 4.67 | | 4.12 | 0 | 2.93±2.55 |  | 0.41 | 0 | 0.93 | 0.45±0.46 | ＜0.001 | 2.72 | |  |
| hsa-miR-490-5p | | | 1.87 | | 2.44 | 3.92 | 2.74±1.06 |  | 0.41 | 1.28 | 1.16 | 0.95±0.47 | ＜0.001 | 1.53 | |  |
| hsa-let-7a-3p | | | 2.65 | | 1.69 | 3.21 | 2.51±0.77 |  | 0.21 | 1.76 | 1.16 | 1.04±0.78 | 0.002 | 1.27 | |  |
| novel-hsa-miR26-5p | | | 1.09 | | 2.62 | 2.32 | 2.01±0.81 |  | 0.41 | 0.96 | 0.69 | 0.69±0.28 | 0.002 | 1.55 | |  |
| novel-hsa-miR181-3p | | | 3.11 | | 0.75 | 2.14 | 2.00±1.188 |  | 4.71 | 4.00 | 5.32 | 4.68±0.66 | 0.012 | -1.23 | |  |
| hsa-miR-1268a | | | 0.93 | | 0.56 | 3.74 | 1.75±1.74 |  | 1.23 | 4.64 | 5.32 | 3.73±2.19 | 0.032 | -1.09 | |  |
| hsa-miR-1307-5p | | | 2.18 | | 0.56 | 1.25 | 1.33±0.81 |  | 0 | 0.48 | 0 | 0.16±0.28 | ＜0.001 | 3.06 | |  |
| novel-hsa-miR200-5p | | | 1.40 | | 0.94 | 0.89 | 1.08±0.28 |  | 0 | 0.16 | 0.46 | 0.21±0.24 | 0.002 | 2.38 | |  |
| hsa-miR-579-5p | | | 0.78 | | 1.69 | 0.54 | 1.00±0.61 |  | 0.21 | 0 | 0.69 | 0.30±0.36 | 0.016 | 1.74 | |  |
| hsa-miR-543 | | | 0.62 | | 0.19 | 1.60 | 0.81±0.73 |  | 1.23 | 3.04 | 2.31 | 2.19±0.91 | 0.025 | -1.45 | |  |
| novel-hsa-miR311-5p | | | 0.47 | | 0.75 | 0.89 | 0.70±0.22 |  | 1.23 | 6.39 | 1.39 | 3.00±2.94 | ＜0.001 | -2.10 | |  |
| hsa-miR-450a-5p | | | 0.93 | | 0.38 | 0.71 | 0.67±0.28 |  | 0.41 | 3.04 | 2.08 | 1.84±1.33 | 0.041 | -1.45 | |  |
| novel-hsa-miR243-5p | | | 0.16 | | 0 | 1.43 | 0.53±0.78 |  | 1.64 | 1.60 | 5.55 | 2.93±2.27 | ＜0.001 | -2.48 | |  |
| hsa-miR-6738-5p | | | 1.40 | | 0 | 0 | 0.47±0.81 |  | 0 | 0 | 0 | 0±0 | 0.016 | 8.87 | |  |
| hsa-miR-1303 | | | 0.16 | | 0.94 | 0.18 | 0.42±0.45 |  | 0 | 0 | 0 | 0±0 | 0.047 | 8.73 | |  |
| hsa-miR-330-3p | | | 0 | | 0.56 | 0.54 | 0.37±0.32 |  | 1.02 | 1.60 | 1.16 | 1.26±0.30 | 0.038 | -1.78 | |  |
| hsa-miR-154-3p | | | 1.09 | | 0 | 0 | 0.36±0.63 |  | 0 | 0 | 0 | 0±0 | 0.047 | 8.50 | |  |
| hsa-miR-329-3p | | | 0 | | 0.19 | 0.89 | 0.36±0.47 |  | 1.23 | 2.08 | 0.46 | 1.26±0.81 | 0.027 | -1.81 | |  |
| hsa-miR-212-3p | | | 0.47 | | 0.38 | 0 | 0.28±0.25 |  | 0.82 | 1.44 | 1.39 | 1.22±0.35 | 0.028 | -2.11 | |  |
| hsa-miR-105-5p | | | 0 | | 0.19 | 0.54 | 0.24±0.27 |  | 0.41 | 0.80 | 2.54 | 1.25±1.14 | 0.018 | -2.38 | |  |
| hsa-miR-365b-5p | | | 0.16 | | 0.19 | 0.36 | 0.23±0.11 |  | 1.23 | 1.92 | 1.16 | 1.44±0.42 | 0.002 | -2.62 | |  |
| hsa-miR-548t-5p | | | 0.31 | | 0 | 0 | 0.10±0.180 |  | 0 | 1.44 | 0.93 | 0.79±0.73 | 0.023 | -2.92 | |  |
| hsa-miR-1304-5p | | | 0 | | 0 | 0.18 | 0.06±0.10 |  | 0.21 | 1.12 | 0.23 | 0.52±0.52 | 0.045 | -3.13 | |  |
| novel-hsa-miR91-3p | | | 0 | | 0 | 0.18 | 0.06±0.10 |  | 0.82 | 1.76 | 0.23 | 0.94±0.77 | 0.001 | -3.99 | |  |
| hsa-miR-1290 | | | 0.16 | | 0 | 0 | 0.05±0.09 |  | 0.21 | 1.28 | 0.23 | 0.57±0.61 | 0.027 | -3.46 | |  |
