# Supplementary material for: MiR-3529-3p from PDGF-BB-induced cancer-associated fibroblast-derived exosomes promotes the malignancy of oral squamous cell carcinoma
Source: Discov Oncol. 2023 Sep 5;14:166. doi: 10.1007/s12672-023-00753-9 (PMC10480386; doi:10.1007/s12672-023-00753-9)
Supplement: Supplementary file 4 — Supplementary file4 (DOCX 28 KB) [file 12672_2023_753_MOESM4_ESM.docx]

**Supplementary table 4 Results of differential top 5 miRNA target gene enrichment in CAFs-Exo and hOMF-Exo**

| Term | Count | % | PValue |
| --- | --- | --- | --- |
| hsa04520:Adherens junction | 31 | 1.35 | 1.23E-09 |
| hsa04110:Cell cycle | 42 | 1.83 | 1.42E-08 |
| hsa04218:Cellular senescence | 47 | 2.05 | 5.87E-08 |
| hsa05203:Viral carcinogenesis | 55 | 2.40 | 2.31E-07 |
| hsa05215:Prostate cancer | 32 | 1.40 | 1.28E-06 |
| hsa05132:Salmonella infection | 61 | 2.66 | 1.62E-06 |
| hsa04068:FoxO signaling pathway | 38 | 1.66 | 3.61E-06 |
| hsa05130:Pathogenic Escherichia coli infection | 50 | 2.18 | 5.76E-06 |
| hsa05220:Chronic myeloid leukemia | 26 | 1.14 | 7.62E-06 |
| hsa04115:p53 signaling pathway | 25 | 1.09 | 1.17E-05 |
| hsa05166:Human T-cell leukemia virus 1 infection | 53 | 2.32 | 1.88E-05 |
| hsa05210:Colorectal cancer | 27 | 1.18 | 2.75E-05 |
| hsa05205:Proteoglycans in cancer | 49 | 2.14 | 3.95E-05 |
| hsa04510:Focal adhesion | 48 | 2.10 | 4.92E-05 |
| hsa04152:AMPK signaling pathway | 33 | 1.44 | 5.68E-05 |
| hsa05213:Endometrial cancer | 20 | 0.87 | 9.92E-05 |
| hsa05161:Hepatitis B | 40 | 1.75 | 1.09E-04 |
| hsa04211:Longevity regulating pathway | 26 | 1.14 | 1.47E-04 |
| hsa04012:ErbB signaling pathway | 25 | 1.09 | 1.82E-04 |
| hsa05131:Shigellosis | 54 | 2.36 | 1.88E-04 |
| hsa05225:Hepatocellular carcinoma | 40 | 1.75 | 2.48E-04 |
| hsa03013:Nucleocytoplasmic transport | 29 | 1.27 | 2.72E-04 |
| hsa05100:Bacterial invasion of epithelial cells | 23 | 1.00 | 2.78E-04 |
| hsa05165:Human papillomavirus infection | 67 | 2.93 | 3.12E-04 |
| hsa01524:Platinum drug resistance | 22 | 0.96 | 3.43E-04 |
| hsa04350:TGF-beta signaling pathway | 26 | 1.14 | 3.74E-04 |
| hsa04810:Regulation of actin cytoskeleton | 48 | 2.10 | 3.81E-04 |
| hsa05216:Thyroid cancer | 14 | 0.61 | 5.77E-04 |
| hsa04722:Neurotrophin signaling pathway | 30 | 1.31 | 6.50E-04 |
| hsa05222:Small cell lung cancer | 25 | 1.09 | 6.59E-04 |
| hsa04210:Apoptosis | 33 | 1.44 | 6.75E-04 |
| hsa04141:Protein processing in endoplasmic reticulum | 39 | 1.70 | 7.34E-04 |
| hsa01522:Endocrine resistance | 26 | 1.14 | 7.35E-04 |
| hsa04151:PI3K-Akt signaling pathway | 69 | 3.01 | 7.56E-04 |
| hsa04010:MAPK signaling pathway | 59 | 2.58 | 9.33E-04 |
| hsa05211:Renal cell carcinoma | 20 | 0.87 | 1.16E-03 |
| hsa04140:Autophagy - animal | 33 | 1.44 | 1.29E-03 |
| hsa05170:Human immunodeficiency virus 1 infection | 45 | 1.97 | 1.32E-03 |
| hsa05167:Kaposi sarcoma-associated herpesvirus infection | 42 | 1.83 | 1.32E-03 |
| hsa05135:Yersinia infection | 32 | 1.40 | 1.61E-03 |
| hsa04066:HIF-1 signaling pathway | 27 | 1.18 | 1.69E-03 |
| hsa05219:Bladder cancer | 14 | 0.61 | 1.72E-03 |
| hsa04216:Ferroptosis | 14 | 0.61 | 1.72E-03 |
| hsa04919:Thyroid hormone signaling pathway | 29 | 1.27 | 1.86E-03 |
| hsa05218:Melanoma | 20 | 0.87 | 2.01E-03 |
| hsa04390:Hippo signaling pathway | 35 | 1.53 | 2.16E-03 |
| hsa03250:Viral life cycle - HIV-1 | 18 | 0.79 | 2.63E-03 |
| hsa04550:Signaling pathways regulating pluripotency of stem cells | 32 | 1.40 | 3.26E-03 |
| hsa01230:Biosynthesis of amino acids | 20 | 0.87 | 3.32E-03 |
| hsa05230:Central carbon metabolism in cancer | 19 | 0.83 | 3.54E-03 |
| hsa05212:Pancreatic cancer | 20 | 0.87 | 3.90E-03 |
| hsa05160:Hepatitis C | 34 | 1.49 | 4.10E-03 |
| hsa04137:Mitophagy - animal | 19 | 0.83 | 4.89E-03 |
| hsa05224:Breast cancer | 32 | 1.40 | 5.04E-03 |
| hsa05169:Epstein-Barr virus infection | 41 | 1.79 | 5.12E-03 |
| hsa04935:Growth hormone synthesis, secretion and action | 27 | 1.18 | 6.03E-03 |
| hsa01521:EGFR tyrosine kinase inhibitor resistance | 20 | 0.87 | 6.12E-03 |
| hsa04934:Cushing syndrome | 33 | 1.44 | 6.16E-03 |
| hsa05226:Gastric cancer | 32 | 1.40 | 6.19E-03 |
| hsa05200:Pathways in cancer | 91 | 3.98 | 6.40E-03 |
| hsa00310:Lysine degradation | 17 | 0.74 | 6.59E-03 |
| hsa05163:Human cytomegalovirus infection | 44 | 1.92 | 7.31E-03 |
| hsa05214:Glioma | 19 | 0.83 | 7.69E-03 |

Note: Some of the lower ranked pathways were omitted.
