# Supplementary material for: MiR-3529-3p from PDGF-BB-induced cancer-associated fibroblast-derived exosomes promotes the malignancy of oral squamous cell carcinoma
Source: Discov Oncol. 2023 Sep 5;14:166. doi: 10.1007/s12672-023-00753-9 (PMC10480386; doi:10.1007/s12672-023-00753-9)
Supplement: Supplementary file 5 — Supplementary file5 (DOCX 26 KB) [file 12672_2023_753_MOESM5_ESM.docx]

**Supplementary table 5. Basic information table of the patients**

| **No.** | **Gender** | **Age** | **Lymph node metastasis** | **Degree of differentiation** | **Diagnosis** | **Sample type** | **Number (cancerous tissue)** | **Number (Paraneoplastic tissue)** |
| --- | --- | --- | --- | --- | --- | --- | --- | --- |
| 1 | Male | 40 | No | Medium | Squamous carcinoma of the oral cavity | Organization | 6 | 2 |
| 2 | Male | 56 | No | High | Squamous carcinoma of the tongue | Organization | 3 | 2 |
| 3 | Female | 56 | No | High | Squamous carcinoma of the tongue | Organization | 2 | 2 |
| 4 | Male | 51 | / | / | Squamous carcinoma of the oral cavity | Organization | 4 | 4 |
| 5 | Female | 46 | / | High | Squamous carcinoma of the lower lip | Organization | 4 | 4 |
| 6 | Male | 68 | Yes | High | Gingival squamous carcinoma | Organization | 1 | 1 |
| 7 | Male | 55 | Yes | High | Squamous carcinoma of the left mandibular gingiva | Organization | 1 | 1 |
| 8 | Male | 63 | Yes | Medium | Squamous carcinoma of the floor of the mouth | Organization | 2 | 2 |
| 9 | Female | 70 | Yes | High | Squamous carcinoma of the tongue | Organization | 2 | 1 |
| 10 | Male | 50 | No | Medium | Highly differentiated squamous carcinoma of the left cheek | Organization | 1 | 1 |
| 11 | Male | 34 | No | Medium | Squamous carcinoma of the tongue | Organization | 1 | 0 |
| 12 | Male | 52 | No | Low | Squamous carcinoma of the oral cavity | Organization | 1 | 0 |
| 13 | Male | 73 | No | High | Squamous carcinoma of the oral cavity | Organization | 1 | 1 |
| 14 | Male | 60 | No | Medium | Squamous carcinoma of the tongue | Organization | 1 | 0 |
| 15 | Male | 40 | No | Medium | Squamous carcinoma of the tongue | Organization | 1 | 1 |
| 16 | Female | 58 | No | Medium | Squamous carcinoma of the tongue | Organization | 1 | 1 |
| 17 | Male | 60 | No | High | Squamous carcinoma of the tongue | Organization | 1 | 1 |
| 18 | Male | 70 | No | Medium | Squamous cell carcinoma of the right cheek | Organization | 2 | 1 |
| 19 | Male | 69 | Yes | Low | Squamous carcinoma of the tongue | Organization | 1 | 1 |
| 20 | Male | 58 | No | High | Squamous carcinoma of the tongue | Organization | 1 | 1 |
| 21 | Male | 59 | No | Medium | Squamous carcinoma of the floor of the mouth | Organization | 1 | 1 |
| 22 | Male | 55 | No | High | Squamous carcinoma of the floor of the mouth | Organization | 1 | 1 |
| 23 | Female | 25 | No | High | Squamous carcinoma of the tongue | Organization | 1 | 1 |
| 24 | Male | 52 | No | High | Gingival squamous carcinoma | Organization | 1 | 1 |
| 25 | Male | 61 | Yes | Medium | Squamous carcinoma of the floor of the mouth | Organization | 1 | 1 |
| 26 | Male | 49 | Yes | High | Gingival squamous carcinoma | Organization | 2 | 2 |
