# Supplementary material for: MiR-3529-3p from PDGF-BB-induced cancer-associated fibroblast-derived exosomes promotes the malignancy of oral squamous cell carcinoma
Source: Discov Oncol. 2023 Sep 5;14:166. doi: 10.1007/s12672-023-00753-9 (PMC10480386; doi:10.1007/s12672-023-00753-9)
Supplement: Supplementary file 6 — Supplementary file6 (DOCX 24 KB) [file 12672_2023_753_MOESM6_ESM.docx]

**Supplementary table 6. Expression statistics of exosome miR-3529-3p in cancer and paracancerous tissues of 20 samples**

| No. | Cancer exosomes | |  | Paracrine exosomes | | *t ^a^* | *P ^a^* |
| --- | --- | --- | --- | --- | --- | --- | --- |
|  | Repeat times | ‾*X±SD* |  | Repeat times | ‾*X±SD* |  |  |
| 1 | 3 | 49.85±59.23 |  | 3 | 1.04±0.34 | 1.431 | 0.289 |
| 3 | 3 | 0.19±0.32 |  | 3 | 1.04±0.35 | -8.674 | 0.013 |
| 5 | 3 | 3.79±0.78 |  | 3 | 1.00±0.09 | 6.633 | 0.022 |
| 7 | 3 | 2.12±0.38 |  | 3 | 1.00±0.10 | 4.941 | 0.039 |
| 8 | 3 | 1.43±0.03 |  | 3 | 1.00±0.10 | 10.857 | 0.008 |
| 9 | 3 | 3.15±0.34 |  | 3 | 1.00±0.08 | 14.075 | 0.005 |
| 10 | 3 | 1.05±0.12 |  | 3 | 1.01±0.13 | 0.651 | 0.582 |
| 13 | 3 | 1.75±0.18 |  | 3 | 1.00±0.06 | 5.722 | 0.029 |
| 15 | 3 | 1.82±0.31 |  | 3 | 1.01±0.19 | 2.944 | 0.099 |
| 16 | 3 | 2.71±0.39 |  | 3 | 1.00±0.04 | 8.536 | 0.013 |
| 17 | 3 | 2.09±0.24 |  | 3 | 1.01±0.13 | 5.631 | 0.030 |
| 18 | 3 | 0.53±0.06 |  | 3 | 1.00±0.04 | -12.414 | 0.006 |
| 19 | 3 | 4.67±0.37 |  | 3 | 1.00±0.10 | 16.841 | 0.004 |
| 20 | 3 | 3.64±0.25 |  | 3 | 1.00±0.06 | 15.850 | 0.004 |
| 21 | 3 | 1.74±0.20 |  | 3 | 1.02±0.26 | 3.216 | 0.085 |
| 22 | 3 | 0.71±0.03 |  | 3 | 1.01±0.18 | -2.560 | 0.125 |
| 23 | 3 | 1.21±0.09 |  | 3 | 1.00±0.03 | 5.837 | 0.028 |
| 24 | 3 | 0.47±0.03 |  | 3 | 1.00±0.06 | -10.200 | 0.009 |
| 25 | 3 | 2.02±0.17 |  | 3 | 1.00±0.10 | 6.744 | 0.021 |
| 26 | 3 | 4.18±0.61 |  | 3 | 1.01±0.17 | 7.929 | 0.016 |
| Sum | - | 4.46±3.21 |  | - | 1.01±0.13 | -3.024*^b^* | 0.001*^b^* |

Note: a:t-test; b:Wilcoxon rank sum test.
