# Supplementary material for: MiR-3529-3p from PDGF-BB-induced cancer-associated fibroblast-derived exosomes promotes the malignancy of oral squamous cell carcinoma
Source: Discov Oncol. 2023 Sep 5;14:166. doi: 10.1007/s12672-023-00753-9 (PMC10480386; doi:10.1007/s12672-023-00753-9)
Supplement: Supplementary file 8 — Supplementary file8 (DOCX 508 KB) [file 12672_2023_753_MOESM8_ESM.docx]

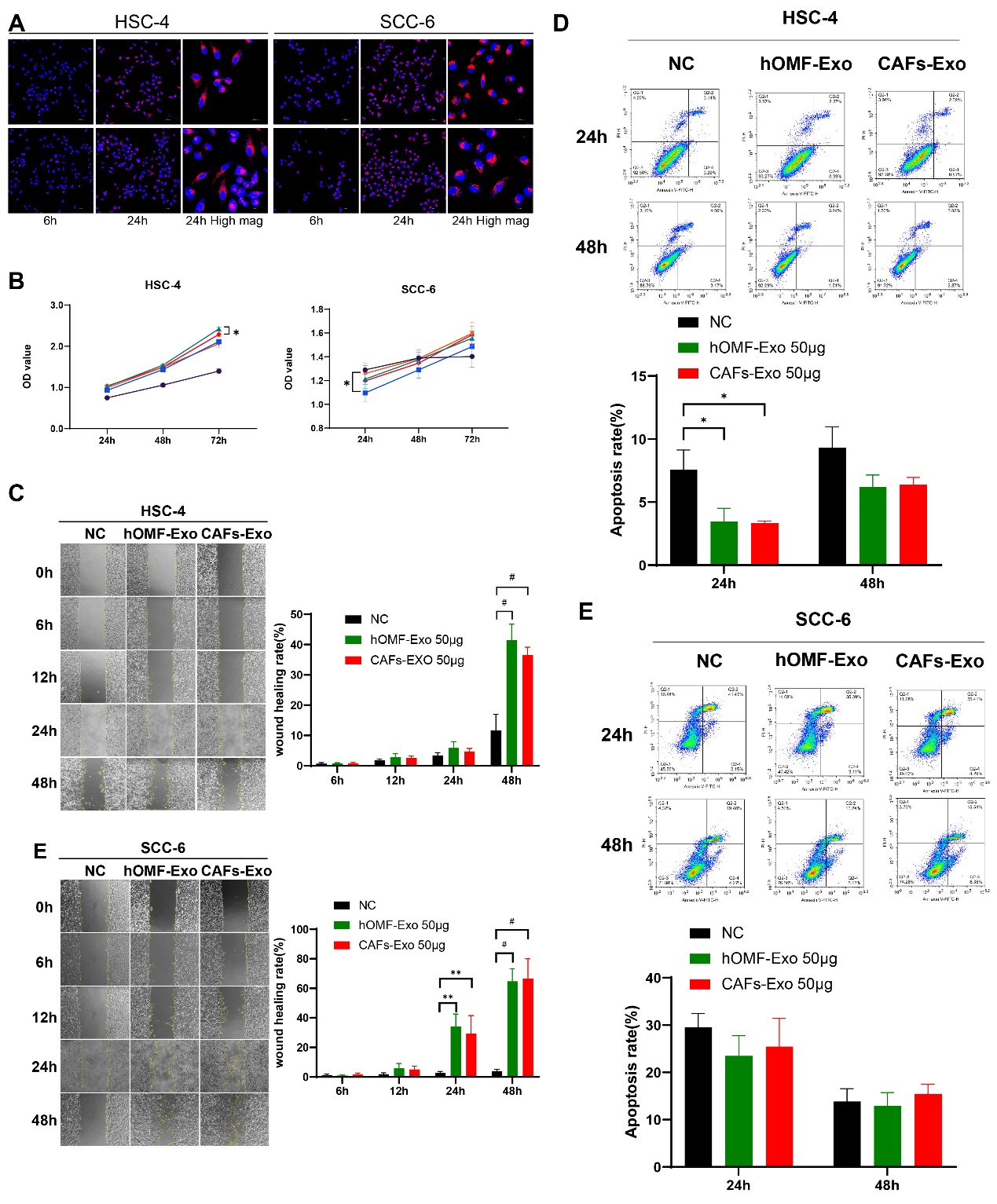


**Figure S1 CAFs-Exo promotes proliferation, migration, invasion, and anti-apoptotic ability in tongue squamous carcinoma cells (HSC-4, SCC-6).**

A: Uptake of exosomes by tongue squamous carcinoma cells, blue fluorescence is DAPI-stained nuclei and red fluorescence is PKH26-labelled hOMF-Exo/CAFs-Exo. B: Changes in proliferation ability after 24h, 48h and 72h of hOMF-Exo/CAFs-Exo treatment in HSC-4 and SCC-6. C: migration of HSC-4 and SCC-6 after 0-48h of hOMF-Exo/CAFs-Exo treatment and quantitative analysis of migration rate. D-E: apoptotic analysis of HSC-4 and SCC-6 after hOMF-Exo/CAFs-Exo treatment for 24h, 48h. **P*＜0.05；** *P*＜0.01；# *P*＜0.001
